# Institutions and Cultural Diversity: Effects of Democratic and Propaganda Processes on Local Convergence and Global Diversity

**DOI:** 10.1371/journal.pone.0153334

**Published:** 2016-04-08

**Authors:** Roberto Ulloa, Celina Kacperski, Fernando Sancho

**Affiliations:** 1 Department of Modern Languages and Literatures, University of Western Ontario, London, Canada; 2 Sport and Exercise Psychology Lab, University of Western Ontario, London, Canada; 3 Departamento de Ciencias de la Computación e Inteligencia Artificial, Universidad de Sevilla, Sevilla, Spain; University of Rijeka, CROATIA

## Abstract

In a connected world where people influence each other, what can cause a globalized monoculture, and which measures help to preserve the coexistence of cultures? Previous research has shown that factors such as homophily, population size, geography, mass media, and type of social influence play important roles. In the present paper, we investigate for the first time the impact that institutions have on cultural diversity. In our first three studies, we extend existing agent-based models and explore the effects of institutional influence and agent loyalty. We find that higher institutional influence increases cultural diversity, while individuals' loyalty to their institutions has a small, preserving effect. In three further studies, we test how bottom-up and top-down processes of institutional influence impact our model. We find that bottom-up democratic practices, such as referenda, tend to produce convergence towards homogeneity, while top-down information dissemination practices, such as propaganda, further increase diversity. In our last model—an integration of bottom-up and top-down processes into a feedback loop of information—we find that when democratic processes are rare, the effects of propaganda are amplified, i.e., more diversity emerges; however, when democratic processes are common, they are able to neutralize or reverse this propaganda effect. Importantly, our models allow for control over the full spectrum of diversity, so that a manipulation of our parameters can result in preferred levels of diversity, which will be useful for the study of other factors in the future. We discuss possible mechanisms behind our results, applications, and implications for political and social sciences.

## Introduction

### Models of culture and social influence

In light of inherent tensions in international integration [1] and a contemporary trend towards cultural policy [2,3], factors that impact cultural globalization and the preservation of diversity have been a recent focus in computational modeling. The question how diversity, i.e. the co-existence of many varied cultures, can be sustained in the face of a growing tendency towards globalization has been explored with various approaches [4–8]. Culture is here construed as the information which is transmitted between individuals in a social manner (such as music, customs, and language). The process of transmission is also known as social influence [9].

Formal mathematical models of social influence illustrated that, when everyone in a network is connected, a global monoculture is inevitable—all cultures converge to a global consensus and become homogenous [4,10,11]. Cultural simulations, among them artificial societies [5,12], have since then been adopted to facilitate the study of patterns of cultural transmission. They have enhanced our understanding of how diversity and global consensus emerge in societies, and how societies can fluctuate between one and the other, exploring these dynamics by introducing various factors to social influence to find ways by which diversity can be preserved.

One example of a social process that has yielded valuable insights is homophily, the principle of "like attracts like": the higher the similarity between two individuals, the more likely they are to influence each other [13,14]. Schelling used this idea to show that a small "dislike" for a dissimilar neighbor could lead to complete segregation in an agent-based model [15,16]. Following this, Axelrod's seminal paper [5] introduced an agent-based model that integrated both, the proposed network structure of previous models [4] and homophily [13], but instead of looking at segregation by movement like Schelling [16], he studied segregation by attitude change, in particular the question: when individuals change their values and opinions based on similarities with each other, do cultures become more alike or more diverse?

He found that cultural diversity emerges and persists under homophily, because groups of agents with similar characteristics grow more similar inside each group, until the groups don't share any common characteristics. Once complete dissimilarity between two groups is reached, they no longer interact. Initial parameters, such as population size, neighbourhood interaction size, and number of cultural features and traits, impacted the emergence of cultural diversity, for example, a smaller population size was conducive to diversity, while an increase in neighbourhood size increased cultural homogeneity [5,17].

In recent research, mass media has been shown to increase cultural diversity when the mass media messages are strong enough, whereas weaker messages were more likely to lead to global homogeneity [18,19]. A change in geography, such as modelling mountains that minimize contact between groups of agents, increased levels of diversity as well [20]. The types of interaction between agents have been also explored: while in Axelrod’s original model, interactions were of dyadic nature, i.e. individuals interacted with and influenced each other on one-on-one basis, Parisi et al [20] and Flache et al [21] implemented multilateral social influence models based on Richardson et al [22], in which agents consider opinions of multiple neighbors around them (instead of just one), before changing their traits.

Finally, Axelrod’s original idea of testing the model against random noise was implemented, in the form of "mutation rates" [20,23], and later, “selection error” [21]. Klemm et al [23,24] introduced various rates of noise into Axelrod's model and found that even at the smallest rate of perturbations, the model quickly destabilized and converged into a monoculture without any diversity, while at a larger rate of perturbation, it devolved into anomie, the complete cultural isolation of each individual from their neighbors [25,26]. The “selection error”, which is based on the assumption of an occasional perception error of a neighbour’s similarity (or dissimilarity), was added to cultural drift as another level of noise, and produced a similar instability [21].

By integrating multilateral social influence with Axelrod’s original postulation of homophily, Flache et al [21] proposed, to the best of our knowledge, the thus far most successful model, facilitating the emergence of cultural diversity and stabilizing Axelrod’s original model [5] against the two sources of noise. It will therefore serve, along with Axelrod's model, as a comparison point in our results.

### Models of institutions

Following cultural drift, limited communication, terrain effects, technology and broadcasting, with the present paper, we would like to introduce a novel question to extend Axelrod’s original model: what role do institutions play in the emergence and resilience of diversity?

First analyses of institutional influence supposed that a diminishing impact of social institutions on values and behavior would increase individualistic tendencies and could, in extreme cases, lead to anomie [26]. Since then, much research into social institutions has investigated their effects in terms of social networks [27,28] and theory of games approaches, such as prisoner’s dilemma and coordination games [29–31]. Very little research has looked at the impact of institutions on culture and its underlying processes of social influence. To our knowledge, only three major projects have used agent-based models in this context: (1) one study showcases how individuals hide their true beliefs in authoritarian regimes (institutions), and how the regimes are affected by this [32]; (2) one platform exists that allows an integration of information repositories, and lets researchers analyze patterns of cultural dynamics [33], and (3) a line of research exists that investigates mass media influence [18,19,34], which can be interpreted as institutional influence and shows several methodological similarities to ours.

The addition of institutions to an agent-based model of cultural patterns, as we propose, can add insight into processes of cultural diversity emergence and resilience by for example analyzing the impact of varying levels of institutional influence and institutional loyalty on culture. Furthermore, we can analyze the way in which individuals and institutions interact with each other inside different political systems, for example through means of democratic processes (like referenda), or organized dissemination of information (like propaganda), and then explore how this impacts the system’s composition.

In agent based models, the idea of "central authorities" has been mostly excluded from the methodology so far. This might be due to the assumption that they can only play the part of central coordinating agents [5,12]. To the contrary, we would like to establish that central authorities and institutions do not denote the same concept. At the center of institutional research lies the exchange between human autonomy (i.e. the agency in human behavior), and social structure (i.e. influences derived by institutions in society) [35]. Axelrod [5] explicitly excludes powerful authorities from his model because of their absolute coordinating impact on culture (where an authority influences individuals’ beliefs and values, but is not in turn influenced in any way). An example of a previous implementation of central authority is the inclusion of geography, such as a mountain range [20]. It impacts agents’ behaviour (by preventing interaction between neighbourhoods of agents), but cannot be impacted by agents itself.

However, authorities are not necessarily absolute. With our present work, we aim at a use of institutions which exert influence on individuals and govern people's behavior and are in turn influenced by individuals, especially in their creation [36]. We speak of institutions in terms of information centers, i.e. mechanisms of political, economic or social interactions [37]. They can be more formal, such as governments, marriage, organized religion, or informal agreements, such as vegetarianism or spiritual beliefs.

In general, space in which shared information is stored does not need to be tangible, but in artificial representations, there is a need to conceptualize a second level of information that lies beyond first level individual interaction patterns. This idea has been previously applied in cultural algorithms (a branch of evolutionary algorithms) in the way of “belief spaces” [38]. Belief spaces inherit cultural knowledge; they are the storage of agents’ shared beliefs, and are updated as those beliefs change. At the same time, belief spaces have an impact on how the agents evolve alongside each other; they impact who interacts with who and who is influenced in what way. In agent-based models, this particular kind of belief space has been termed a "cultural repository" [33].

**Fig 1:**
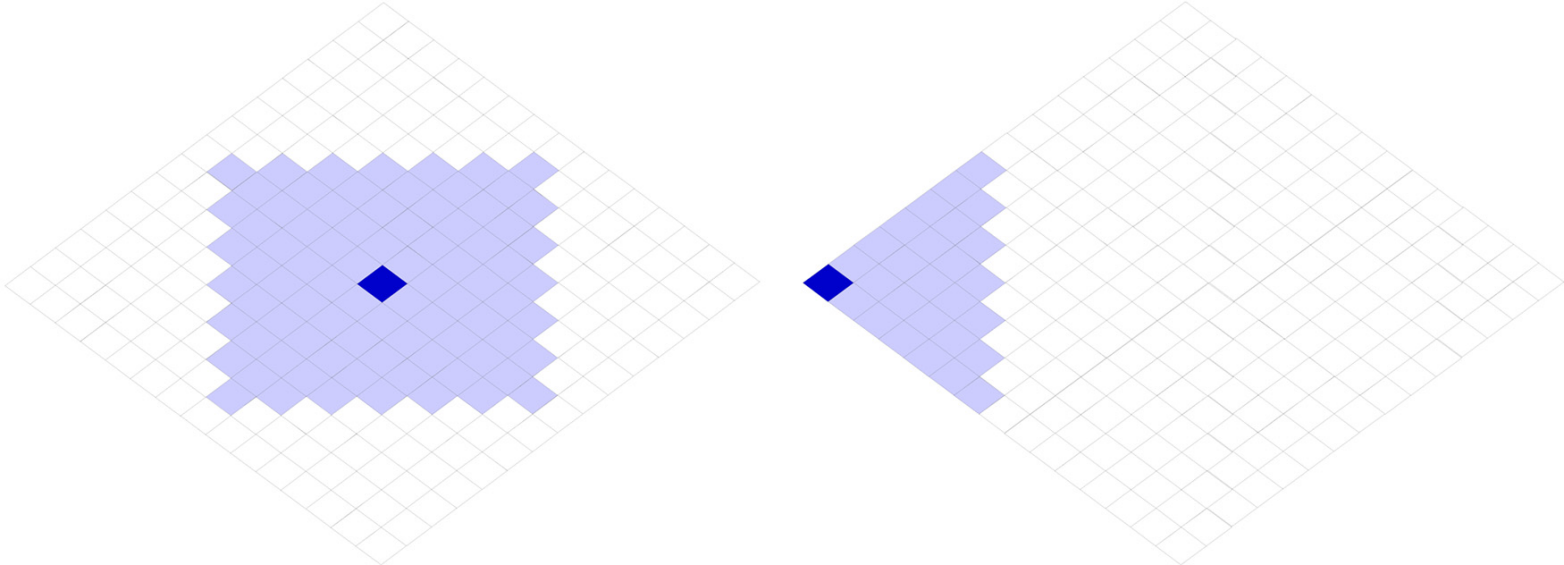
Model of institutions, Romeo and Juliet example. "R" represents agent Romeo, "J" represents agent Juliet, "M" and "C" their respective houses, Montague and Capulet. Agents from one cultural region (e.g. yellow, blue) are connected to the institution that they belong to (also colored yellow and blue).

In order to illustrate the relationship of these two levels of information storage, let us assume, for example, that Romeo interacts with Juliet, discussing the value of certain types of music. In Axelrod’s model [5], there is only one level: through homophily and social influence, Romeo can be convinced by Juliet that salsa music is better than hard rock, and Romeo and Juliet would then share a common "trait". In our model, both Romeo and Juliet still interact on an individual level, but they also have a belief space that represents the two different institutions that they belong to, for example their respective familial units, House Montague and House Capulet (Fig 1). When Romeo interacts with Juliet, he is not only aware of their interpersonal similarity and their own traits, he is also pressured by how representative his institution (i.e. his family) is of him, and how much influence this has on him. The level of *institutional influence* that Romeo perceives can prevent him from liking salsa music. He needs to check whether his family approves of salsa music, and if it doesn’t, whether his homophily with Juliet is strong enough to ignore his family.

If Romeo does change his trait, because he likes Juliet better than his family, he can then also choose to change his institution, i.e. see if becoming a Capulet will suit him better than being a Montague, as Juliet’s family, the Capulets, might be more representative of who he is than his own family. This choice will depend on Romeo's *loyalty* towards his family, i.e. how willing he is to give up his family name and connections.

The different levels of institutional influence and agent loyalty can be exemplified by different types of institutions. For example there are institutions that promote strong identification and exert a lot of influence, such as families or nationalities, or those that do less so, such the school one went to, or the TV channels one watches. Individuals can feel varying levels of loyalty to their institutions as well, for example when someone is part of a political party because it has always been this way in their family (conservatism), or when social punishment is normative (e.g. some familial structures or religious organizations).

With all these considerations in mind, we would like to propose our initial research questions: How is the diversity of a system impacted by varying amounts of influence that institutions exert on individuals? How is the system impacted by varying amounts of loyalty that institutions demand from their followers? And how does the inclusion of institutional influence and agent loyalty to an agent-based cultural dissemination model compare with results obtained by Axelrod [5] and Flache et al [21]?

### Referendum and propaganda

Our previous research questions look at the impact of various levels of influence and loyalty on cultural diversity in a way in which institutions only prevent possible cultural changes (the influence is indirect). An additional focus of our work are the two directions in which social influence can function between low level interactions (individuals) and high level interactions (institutions) [35,36], and how this impacts cultural dissemination; on one hand, influence can be exerted in a bottom-up trend, on the other hand, it can be exercised in a top-down manner. Thus, conceptually, the processes of institutional influence we propose can be understood as a feedback loop of information.

These two forms of direct influence have found translation into forms of governance employed in political systems. Common bottom-up influences upon institutions are voting, and mechanisms of direct democracy such as referenda or plebiscites [39], while a common form of top-down influence by institutions is the use of information dissemination, such as campaign advertising or propaganda [40]. Some previous agent-based models have looked at the impact of a feedback loop of influence on cultural diversity and have related this procedure to mass media coverage on a global population level [18] or differentiated by predetermined local neighborhoods [19]. However, those models do not differentiate between top-down and bottom-up processes, and they lack a validation against noise levels, which have been shown to greatly impact system stability [23].

By reason of this, we would like to propose another line of investigation, subdivided into three specific research questions. We will first individually explore a bottom-up process, in which the institutions adapt their traits towards population majority beliefs, similar to the execution of referenda, and see how this process impacts the composition of institutions, their influence, and therefore, the emergence and persistence of diversity within the system. Then we will explore a top-down process in the same manner, in which individuals adapt their traits due to institutional pressures, similar to the employment of propaganda. Lastly, we will combine the two in one system to show the effects of the feedback loop of social influence. We will also see how a variety of frequencies of occurrence of these processes affect system stability under noise.

## Methods

### Agent-based models

Agent based models are a popular tool for empirical testing of realistic concepts in complex systems. Initial models were common in economy and biology [41–43] and have since then permeated many knowledge fields ranging from psychology to physics. In the social sciences, Epstein & Axtell popularized their use with their sugarspace model [12].

Models commonly include a two-dimensional grid, which serves as the “world”. It is inhabited by agents, which can be interpreted as individuals, tribes or villages/towns. How agents interact with each other and their world environment is based on the rules of any given model. This type of abstraction allows a representation of a variety of patterns and ideas, such as cultural patterns, while not applying it to any specific, named culture.

**Fig 2:**
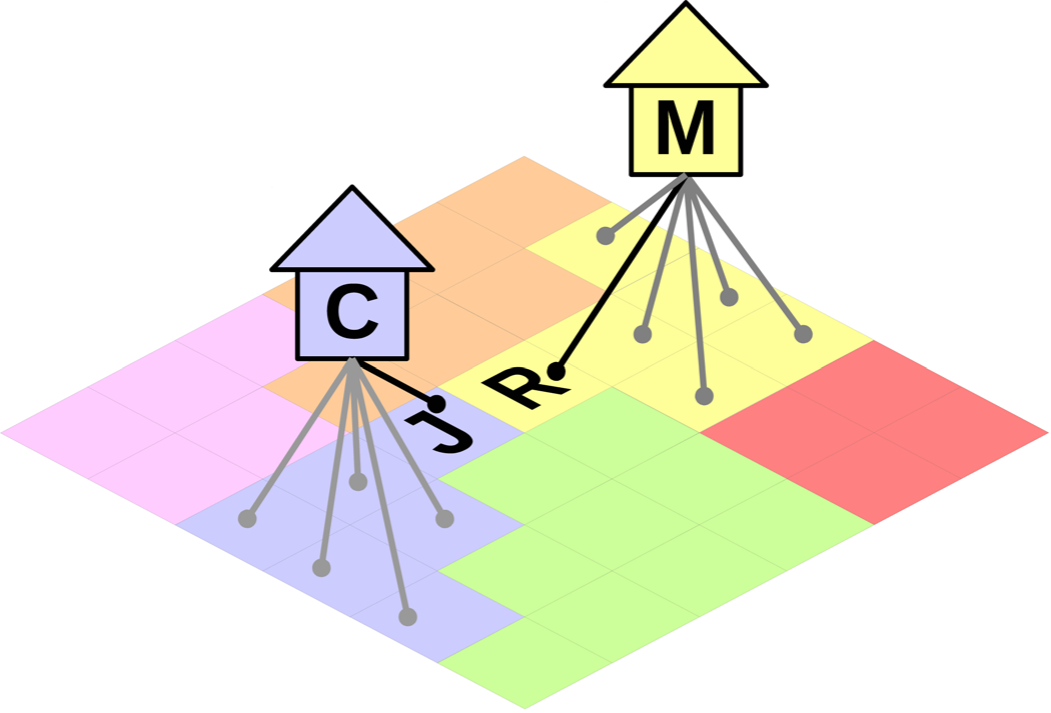
Two possible von Neumann neighbourhoods with distance 6. Two example neighbourhoods on a 15x15 grid. Left: central agent (dark blue) with 84 neighbouring agents (light blue) that it can interact with. Right: border agent (dark blue) with 27 neighbouring agents (light blue).

In our model, we follow and expand the arrangement of Axelrod’s original converging diversity model [5] with the following units:

1. Individual agents: Each individual agent is an autonomous entity that holds a certain amount of information, its culture. In the simulation, this culture is represented by a string of features, such as for example music, cuisine, language, etc. Each agent exhibits a preference on each feature, which is called a trait; an agent might like salsa music, Mexican food and Spanish language. In simulations, these traits are represented by integer values. Based on Flache et al [21], we used 5 features and 15 possible traits per feature. An agent ***a*** can be represented as follows: [2, 4, 12, 9, 14], in which ***a***_***f***_ refers to the trait on feature ***f*** for agent ***a***.
2. Institutional repositories: Each institution is a second layer "agent" that represents a belief space. It is denoted by a string of features and their trait exhibits are based on integer values, exactly as individual agents. However, unlike individual agents, institutions are not attached to a grid and they also can have empty features (features without an assigned trait), internally represented with -1. An agent ***a*** can only belong to one institution at a time, denoted ***i***_***a***_, and it follows that ***i***_***af***_ will represent the trait for feature ***f*** of ***a***'s institution. Every individual agent is initially associated with an institution with all empty features. The agent can then, in interaction and agreement with another agent, overwrite one feature. An agent can also switch their institutional association to be associated with a different institution instead.
3. Grid environment: Agents are arranged on a two-dimensional lattice. We follow Flache et al [21] in selecting three varying sizes for the lattice, 10x10, 32x32, and 100x100, making up world populations of 100, 1024 and 10000 agents. Although human populations are generally much bigger, 10000 agents can be a representation of a town, or, according to Axelrod [5], each one agent can be representative of one village. By studying different sizes, we can observe whether our patterns will stay consistent across various population sizes (bigger populations than 10000 agents were difficult to study due to high computational demands). Neighborhoods are defined as the areas within which agents can interact. We define a Von Neumann neighborhood within a distance of 6, meaning each agent can interact with a maximum of 84 agents (Fig 2).
  The environment is represented as a non-toroidal world, so agents have fewer neighbors when they are closer to the borders. Agents that share the exact same trait combination on all their features and are located next to each other in the grid are considered to be of the same culture. All agents that belong to the same culture determine a "cultural region". Diversity is defined as at least two existing trait variations, e.g. cultural regions, existing at the same time. Anomie indicates that there are as many cultural regions as there are agents; conversely, complete globalization denotes that there is only one cultural region.
4. Rules: The model integrates dynamical rules which allow agents to interact based on probabilities associated to cultural similarity. Cultural similarity is the number of cultural traits that are equivalent on two vectors of agents or institutions.

There are three types of probabilities working in the model: (1) agent similarity, which is the similarity between two agents (i.e. homophily); (2) internal institutional similarity, which exists between an agent and its own institution, and (3) external institution similarity, which exists between an agent and the institution of a neighbor.

Institutions impact agent interactions based on these cultural similarities, and based on two factors: institutional influence and agent loyalty. Agents impact institutions when they create institutions, or when they join an existing institution and the institution’s "active feature" is empty. An "active feature" denotes the one particular feature that is being discussed when two agents interact.

Table 1 presents an overview over the most important rules that we are including in our simulations, and the combinations in which they were implemented in previous research.

**Table 1:** Overview over previous models and their included parameters, and our model.

Model	Homophily	Perturbation Mutation	Perturbation Selection Error	Multilateral Social Influence	Institutions
*Axelrod 1997*	Yes	No	No	No	No
*Klemm 2003*	Yes	Yes	No	No	No
*Flache 2011*	Yes	Yes	Yes	Yes	No
*Ours*	Yes	Yes	Yes	No	Yes

The first column identifies models by author. The second to sixth column indicate different rules that have been tested in the different models.

Firstly, Axelrod's original model [5] generated cultural diversity by using homophily to regulate dyadic social influence. Secondly, Klemm et al [24] showed that various rates of mutations destabilize Axelrod's cultural diversity, with low rates converging the model into monoculture while higher rates led to anomie. Finally, Flache et al [21] introduced multilateral social influence to Axelrod's homophily (based on work by Parisi et al [20]).

We are adopting the same conceptualization of social influence (directed by homophily) as Axelrod [5]. Homophily of agents ***a*** and ***n*** determines whether an interaction occurs or not, based on the number of equivalent traits in both agents, so we can use the following function of similarity (***Sim(a*, *n)***) to calculate the homophily between agents:

$$Sim\left(a,n\right)=\frac{1}{F}\overset{F}{\underset{f=1}{\sum^{\text{​}}}}\delta \left(a_{f},n_{f}\right)$$ (1)

Here, ***F*** is the total amount of features and the ***δ(i*,*j)*** function refers to the Krockener delta:

$$\delta \left(i,j\right)=\{\begin{array}{ll} 1 & i=j\\ 0 & i\neq j \end{array}$$ (2)

We will conceptualize noise in the same way Flache et al [21] did: an instance of mutation [23] occurs when the trait of an agent, after a possible interaction, is randomly selected and set to a new feature. A mutation occurs with probability ***m***. An instance of selection error [21] occurs after the initial interaction outcome has been decided based on homophily, and it reverses the initial decision to interact (or not). A selection error occurs with probability ***s***. In order to simplify the study we will keep ***m = s*** across all the experiments, as Flache et al [21] did.

We also decided to integrate the selection error into the homophily rule, resulting in one formula, which we call Perceived Homophily (***PH(a*,*n)***):

PH(a,n)=(1−s)Sim(a,n)+s(1−Sim(a,n) (3)

Our Perceived Homophily rule is equivalent to Flache et al's approach of applying homophily first, and selection error second [21].

Finally, our response variable will be the number of cultural regions remaining after the final agent interaction iteration. We decided on a different response variable than Flache et al [21] due to the different outcomes that we are analyzing. Flache et al used the "normalized size of the largest region" in order to observe how noise affects the tendency towards a monoculture or globalization in a system, with the assumption that this measure does reflect diversity, without the need to explicitly state the number of cultures. We have chosen to examine the number of cultures, since we are interested in cultural diversity per se as it emerges and is preserved by manipulation of different institutional factors, so our measure is more fitting and expressive of our purposes. It will be normalized through division by the total number of agents (denoted ***N***) in the given simulation, to best showcase similarities across different population sizes.

$$response\;variable=\;\frac{total\;number\;of\;cultural\;regions}{N}$$ (4)

For a comparison of the two response variables in question and how they affect our results, please refer to S6 File.

### Baseline: Models of institutional influence and loyalty

The main purpose of our investigation of institutions is their impact on cultural patterns reflected in Axelrod’s model of dyadic social influence. For our baseline model we integrate institutional influence into our combination of dyadic social influence with homophily [5], mutation [23,24], and selection error [21]. For this intent, we use the institutional influence function (***Inf(a*,*n)***):

$$Inf\left(a,n\right)=\frac{\alpha \cdot Sim\left(a,i_{a}\right)}{\left(1-\alpha \right)\cdot Sim\left(a,n\right)+\;\alpha \cdot Sim\left(a,i_{a}\right)}\;,\;\text{where }\alpha \;\epsilon \;\left[0,1\right]$$ (5)

Institutional influence is exerted as a combination of agent similarity, (***Sim(a*,*n)***) and institutional similarity ***Sim(a*,*i***_***a***_***)***,. We define institutional similarity in the same way as the original homophily formula of similarity. As the formula expresses, the probability of trait change decreases as the agent’s similarity to its own institution increases. Moreover, the alpha parameter (***α***) controls the amount of institutional influence. This same value is applied to all the agents in the simulation. The bigger the ***α***, the more importance agents give to their institutions, and therefore the less likely it is that a trait change will occur.

Institutional influence, ***Inf(a*,*n)***, is applied only when there is an institutional conflict, i.e. a situation in which the agent ***a*** currently holds the same trait as its own institution, ***i***_***a***_, but the "active trait" (i.e. the agent’s to-be-adopted trait by social influence) is different from the trait that the institution ***i***_***a***_ holds. The agent is "being tempted" into dissimilarity from its own institution, and the institution is exerting its influence to stop this from happening. In the case where there is no institutional conflict, the formula for Perceived Homophily ***PH(a*,*n)*** is used instead, and no institutional influence is exerted.

Because of constant interactions with neighbors, an agent’s cultural vector can turn out to be more similar to its neighbor’s institution than its own. In this case, after the interaction with its neighbor, the agent checks if a change of institutions is favourable. We determine whether an agent ***a*** will remain loyal to its own institution ***i***_***a***_ when confronted with the institution ***i***_***a***_ of the neighbor ***n*** by applying the agent loyalty function ***Loy(a*,*n)***:

$$Loy\left(a,n\right)=\frac{\alpha \prime \cdot Sim\left(a,i_{a}\right)}{\left(1-\alpha \prime \right)\cdot Sim\left(a,i_{n}\right)+\;\alpha \prime \cdot Sim\left(a,i_{a}\right)}\;,\;\text{where }\alpha \prime \;\epsilon \;\left[0,1\right]$$ (6)

The ***α'*** parameter controls the agent's loyalty to its current institution and applies identically to all agents: the higher the ***α'*** parameter, the higher the agent's loyalty towards its original institution, and the less likely an institutional change. We introduce the agent’s similarity to the neighbor’s institution, ***Sim(a*, *i***_***n***_***)***, into the denominator's function, so that the probability of institutional change increases as the agent’s similarity to its neighbour’s institution increases.

### Extensions: Models of referenda and propaganda

After investigating the stability and diversity values of our baseline model, we will investigate the influence processes between agents and institutions in two ways.

The first extension is a bottom-up process that resembles a democratic process, e.g. a referendum. Agents can influence their institutions with the intention of aligning the institution towards the agents' traits. When many individuals in a population manifest disagreement with their institution’s current stance on a particular issue, they can force the institution to change towards the popular opinion. In our model, we adopt this extension by selecting one institutional trait that is the least popular among the given population at the time; then we allow agents to “vote” to change it to that trait which the majority approves. We can control the prevalence of democracy by specifying the number (***X***) of interaction opportunities that each agent has with another agent before they are allowed to act together in a voting process. This is called the frequency of democracy, ***fd***, and it is the reciprocal of ***X***, i.e. ***1/X***.

The second extension is a top-down process that resembles a propaganda campaign of an institution, by way of dissemination of its cultural traits with the intention of aligning more of the agents' traits towards itself. In our model, we adopt this extension by allowing the institution to try and push its traits onto each of its affiliated agents. Whether an agent allows the propaganda to change its trait depends on how similar this agent ***a*** already is to its own institution, i.e. ***Sim(a*, *i***_***a***_***)***. We control the prevalence of propaganda the same way we did for democracy, i.e. by a certain number (***Y***) of interaction opportunities between agents, and define the frequency of propaganda, ***fp*,** as the reciprocal of ***Y***, i.e. ***1/Y***.

We will also combine the two extensions in our last model to analyze the results of a combination of both bottom-up and top-down processes together, in the form of a feedback loop of institutional influence.

We summarize all formal rules which we are adopting for our six model variations in Table 2. Rules are constructed based on Axelrod's model (A), to which we add our baseline (B, institutional influence and agent loyalty), and which we then extend by adding a democratic process (D, e.g. a referendum), or a propaganda process (P, e.g. advertisement campaigns), or both combined.

**Table 2:** Formal rules of presented models.

Step	A	B	D	P
1. At random, pick one agent ***a*** and one of its neighbors ***n*** from ***a***’s possible neighbors, as defined by a radius ***r***	X	X	X	X
2. Randomly select a feature f* (of those features that have differing traits of **a** and **n**). Then, select t* = n_f*_	X	X	X	X
3. **(Institutional conflict)** If the current trait of the agent's institution ***i***_af*_ (1) is not undefined (***i***_af*_ ***≠ -1***), and (2) it is equal to the agent's existing trait ***a***_f*_ (i.e. ***i***_af*_ ***= a***_f*_), and (3) if the institution's trait ***i***_af*_ is different to the to-be-adopted trait t* (***i***_af*_ = t*), then		X	X	X
3.1. **(Perceived Homophily + Institutional Influence)** Agent **a** accepts the trait t* for f* with a probability of trait change ***P***_***tc***_ equal to ***Inf(a*, *n)***		X	X	X
3.2. **(Agent loyalty)** If agent **a** accepts the trait t*, then ***a*** changes its institution to ***i***_***n***_ with a probability of institutional change ***P***_***ic***_ equal to ***Loy(a*, *n)***		X	X	X
3.3. If the agent ***a*** changes its institution to ***i***_***n***_, and if ***i***_***n***_ does not yet have a trait on the selected feature f*, then assign t* to ***i***_***nf***_.		X	X	X
4. **(Perceived Homophily)** If the conditions in the previous step were not met or for a model without institutions; then the agent ***a*** accepts the trait t* with a probability of trait change **P**_**tc**_ equal to ***PH(a*, *n)***	X	X	X	X
5. **(Mutation)** With probability ***m***, randomly change one of the features of agent **a** to randomly selected trait	X	X	X	X
6 **(Democracy)** After ***fd × N*** (***N*** is the population size) repetitions of steps 1 to 5, initiate a Democratic process. For each institution ***i***:			X	
6.1 A subset ***D*** containing all agents belonging to ***i*** is created.			X	
6.2 All agents in ***D*** cast a vote containing their current trait for each of their features. A voting matrix ***V***, is generated, where ***V***_***ft***_ corresponds to the number of votes that trait ***t*** received for feature ***f***, i.e. ***V***_***ft***_ ***= Σ***_***d∈ D***_ ***δ(d***_***f***_**, *t)***			X	
6.3 A matrix **W** is defined by ***W***_***ft***_ ***= V***_***ft***_***—V***_***fc*,**_ where ***c*** is the current trait for the feature ***f*** of the institution ***i***. This matrix holds the differences on popularity (votes) between the current traits of the institution and their alternatives.			X	
6.4 Create a subset ***FT*** of pairs ***(f*, *t)*** in which ***W***_***ft***_ is maximal in ***D*** and bigger than zero. This subset contains the traits that comprise the biggest differences between each institution and its agents.			X	
6.5 Randomly select a pair (f*,t*) from ***FT*** and replace ***i***_***f***_ with t*			X	
7. **(Propaganda)** After ***fp × N*** (***N*** is the population size) repetitions of steps 1 to 5, initiate a Propaganda process. For each institution ***i***:				X
7.1. A subset ***P*** containing all the agents belonging to ***i*** is created. For each agent ***a*** in ***P***:				X
7.1.1 Calculate the similarity between agent ***a*** and ***i***, and set this as the probability of trait change, i.e. ***Ptc = Sim(a*, *i)***				X
7.1.2. For each feature ***f*,** change ***a***_***f***_ ***= i***_***f***_ with probability of trait change ***Ptc***				X

The first column indicates the rules inherent in each step of a given model. The second to fifth column indicate the model to which they apply (A: Axelrod, B: Our Baseline, D: Democracy extension, P: Propaganda extension). The star symbol (*) indicates a specific value of a variable.

### Experimental Design

We are exploring the effects of institutional influence and agent loyalty, and comparing some results with Axelrod and Flache et al by replicating their models with our code. We are also integrating democratic processes, propaganda processes, or both together into simulations. We are therefore presenting the results of six different experiments, called experiments A to F. For all six experiments, we hold certain factors constant. Results are presented across the three chosen population sizes (10x10, 32x32, 100x100), and for six chosen levels of noise (10^-n^, where e n ∈ {6,5,4,3,2,1}). Agents hold 5 features (F), 15 traits (T), and between 27 and 84 neighbors (depending on their position on the grid and a Von Neumann neighborhood of 6). All these values were chosen based on Flache et al [21]. The number of agent interaction iterations is set at 100000 possible interactions on average per agent, which has previously been shown to be the number at which a population can be expected to have converged to a stochastically stable state (p.218 in [5]). Please find results that validate an equilibrium state after 100000 interactions for our model in S5 File. Since each simulation is non deterministic (e.g. agent traits, social and institutional influences depend on probabilities), every run can, and often does, produce different results even when undergoing the exact same treatment (i.e. when we run the simulation with the same combination of factors levels). Therefore, we repeat each treatment 50 times, and our response variable is the average of the 50 results, with each result being one normalized number of cultural regions after the last iteration.

For an overview over the contrasting factors for experiments A to F, please refer to Table 3. For the sake of readability, unless explicitly specified by the formula (***Inf(a*,*n)***), institutional influence will from now on be referred to as the alpha parameter (***α***) and agent loyalty, (***Loy(a*,*n)***), as the alpha prime parameter (***α'***). Notation to identify specific models will be population/α/α', for example 10x10/0.8/0.95 for the smallest population with institutional influence of 0.8 and agent loyalty set at 0.95.

**Table 3:** Contrasting factors for experiments A to F.

	A	B	C	D	E	F
Model (M)	Ours	Axelrod's, Flache's, Ours	Ours	Ours + Democracy	Ours + Propaganda	Ours + Democracy + Propaganda
Institutional influence (α)	[0.5, 1[	Axelrod's: N/A, Flache's: N/A, Ours:0.8, 0.9	10x10: 0.85, 32x32: 0.8, 100x100: 0.75	10x10: 0.85, 32x32: 0.8, 100x100: 0.75	10x10: 0.85, 32x32: 0.8, 100x100: 0.75	10x10: 0.85, 32x32: 0.8, 100x100: 0.75
Agent loyalty (α′)	0.5	0.5	0.05, 0.5, 0.95	0.5	0.5	0.5
Frequency of democracy (fd)	N/A	N/A	N/A	1/10,1/100, 1/1000	N/A	1/10,1/100, 1/1000
Frequency of propaganda (fp)	N/A	N/A	N/A	N/A	1/1, 1/3, 1/5	1/1, 1/3, 1/5

The first row identifies the letters assigned to each of our experiments (A to F), the second row is a brief description of the models. The first column identifies all the factors involved in each experiment. Remaining columns display the values that were chosen in the respective models.

For the first three experiments, A, B and C, we are exploring the effects of varying values of institutional influence and agent loyalty on cultural diversity: for experiment A, we manipulate institutional influence ***α***, from 0.5 to 1.0 to test its impact on cultural diversity, while holding agent loyalty, ***α'***, constant at 0.5. Experiment B replicates Axelrod's model [5] and Flache's model [21] to directly compare their results with the values that achieved the most similar results to Flache et al in experiment A, i.e. ***α*** = 0.8 and ***α*** = 0.9.

For experiment C, we are manipulating agent loyalty by applying extreme values of 0.05 and 0.95, and comparing this to the 0.5 baseline value from experiments A and B. We also select three different ***α*** values for the three given populations: 0.85 for 10x10, 0.8 for 32x32, and 0.75 for 100x100. We do this in order to showcase that the subsequent results are not dependent on one particular value of influence, and because they provide some variance in initial cultural diversity (higher for smaller populations, and lower for bigger populations) while not being extreme, i.e. too near of either globalization or anomie. This moderate rate is valuable in our study of others factors; if the institutional influence chosen induces too much diversity to start with, we might not be able to see whether loyalty values increase diversity as well.

Experiments D and E manipulate the frequency of democratic and of propaganda processes by changing the number of interaction opportunities that each agent has with another agent; for democracy, these values are set at 1/10,1/100,and 1/1000, for propaganda at 1/1,1/3 and 1/5.

Experiment F combines democracy (at frequency 1/10,1/100 and 1/1000) and propaganda (at frequency 1/1,1/3,and 1/5) with their above described frequencies, and investigates the interaction of both.

## Results

The following section is subdivided into two parts: we will first explore and discuss the effects of our two main model parameters, institutional influence and agent loyalty, on diversity, and compare them against results obtained in two previous models, i.e. Axelrod's and Flache's, in experiments A to C. We will then in the second section provide the results of our democratic model, of our propaganda model, and the integration of both democracy and propaganda in one model, i.e. experiments D to F, and discuss the implications inherent in those extensions.

### Results: Experiment A to C

#### Experiment A: Institutional influence

In our first experiment, we manipulated institutional influence while holding agent loyalty constant (***α'*** = 0.5). We explored all institutional influences from 0 to 1.0, but we found no relevant results for values for ***α*** < 0.7. For those values, the model was highly sensitive to low levels of noise, and, for populations of 10x10 and 32x32, we found very small differences in cultural diversity, even in a configuration with almost no noise.

**Fig 3:**
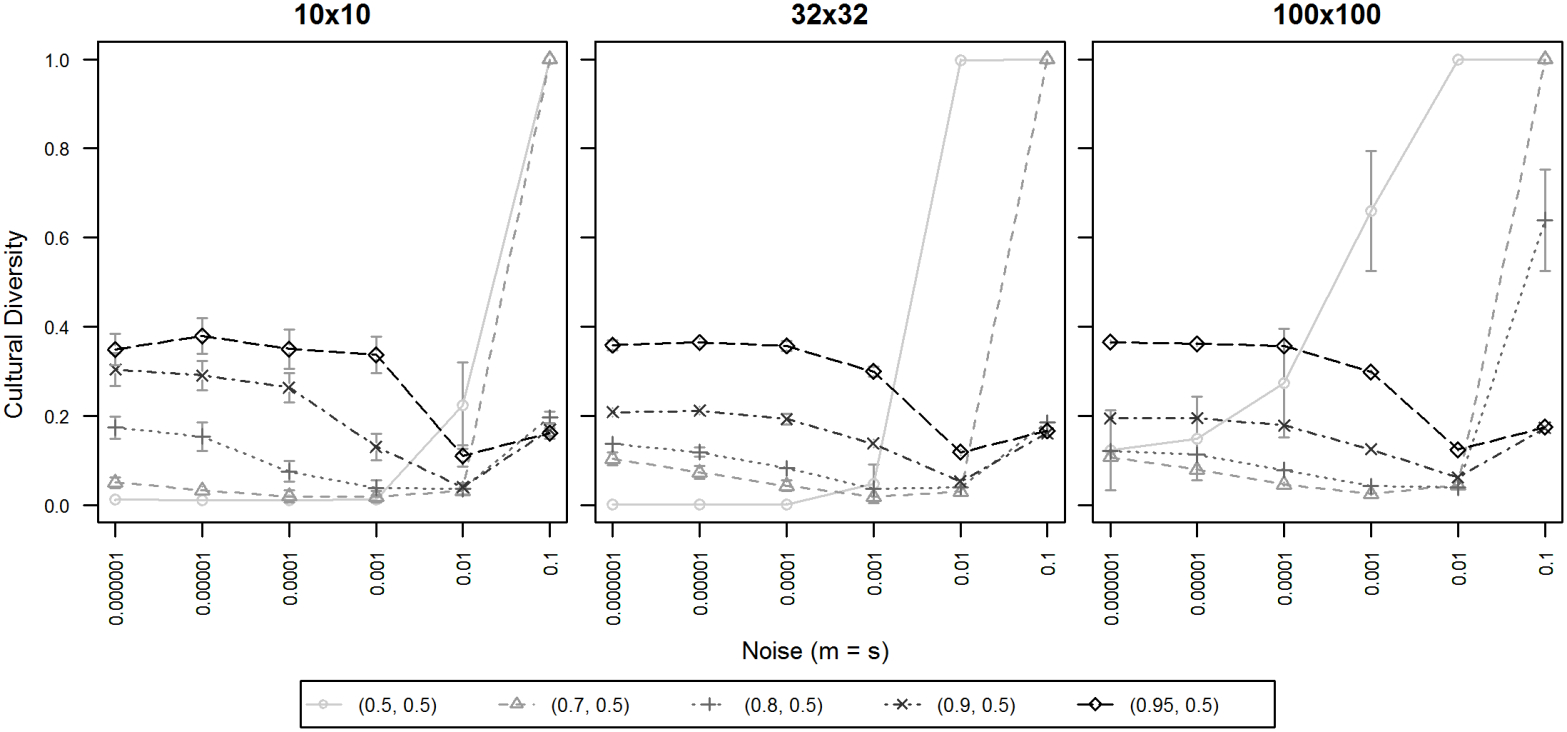
Cultural diversity for varying levels of institutional influence. X-axis displays levels of noise; Y-axis displays normalized cultural diversity. Each line symbol denotes one ***α*** of institutional influence. 95% confidence intervals are displayed only when exceeding the size of the line symbol. Data points are averages of 50 repetitions per territory with 100,000 iterations per agent.

In Fig 3 we present values for which we found relevant results of institutional influence, i.e. ***α*** = 0.5, 0.7, 0.8, 0.9 and 0.95, and we show how they affect our three chosen population sizes.

Based on the graphic representation of our results, we can identify that cultural diversity varies greatly depending on institutional influence, in particular when levels of noise are very low (< = 0.001). In this range of noise, we found that the more institutional influence was exerted, the more diversity was found across the entire population. While for low noise (< = 0.01), high values of alpha (***α*** > = 0.7) did still sustain diversity, we observed that under the highest level of noise (0.1), only higher values of alpha (***α*** > = 0.8) were able to sustain diversity. Lower values of noise (< = 0.7) induced a state of anomie.

These results are similar for all three population sizes, and similarity increase with an increasing α value. We found no significant effect that would suggest differences based on population size, with F(2, 882) = 0.17, p = 0.85, when we input all population sizes and noises as factors in an ANOVA and calculated differences for ***α*** = 0.95. When we compared 32x32/> = 0.7/0.5 and 100x100/> = 0.7/0.5 in an ANOVA (with noise < = 0.01), we also found no significance effect between them, with F(1, 1960) = 1.41, p = 0.23. For further details on the performed ANOVA calculations, please refer to S1 File.

Since we normalized the number of cultural regions by population, a proportional diversity by population means that calculated by absolute values, on average, the bigger the given population, the more cultural regions remain after the last interaction. Consequently, on average, reported normalized cultural regions tend to be of the same size. Table 4 gives an overview over the absolute number of cultures obtained for our models at the lowest level of noise (0.000001). Especially for the higher alphas (***α*** > = 0.8), a linear relationship is visible, as we perceived an increase of cultural regions by a factor of 10, in linear relation with the increase of the population size by a factor of 10.

**Table 4:** Number of cultures and institutions (cultures / institutions) per population size over alpha values 0.5 to 0.95.

	100 (10x10)	1024 (32x32)	10000 (100x100)
0.5	1.14 / 10.9	1.02 / 48.00	1219.46 / 102.98
0.7	4.98 / 7.94	106.12 / 76.98	1073.12 / 691.68
0.8	17.32 / 8.28	139.88 / 55.50	1210.38 / 462.92
0.9	30.38 / 6.92	212.98 / 31.58	1944.98 / 243.5
0.95	34.82 / 6.32	367.28 / 41.98	3647.70 / 370.98

Averages of 50 repetitions, after 100000 iterations per agent, with noise level at 0.000001.

Table 4 also displays the total number of institutions and its similar linear relationship with population size. For all data, there was a strong positive correlation between the population and the number of institutions, r = 0.53, p < 0.0001. This correlation increased for higher alphas, e.g. for ***α*** > = 0.9, r = 0.84, p < 0.0001. At this point (α > = 0.9), there was a very strong positive correlation between the number of cultures and the number of institutions, r = 0.93, p < 0.0001. We can observe in Table 4 that for most data points, more cultures than institutions exist, with the only exceptions at lower alphas (i.e. at 32x32/0.5/0.5, and at 10x10/0.7/0.5).

#### Experiment B: Replication of Axelrod's and Flache's models

In order to compare the results of our first experiment with other models, we replicated Axelrod's [5] and Flache's models (experiments 1 and 3, p.978 & p.984 in [21]), using an implementation with our own code. For a detailed comparison of the implementations, please see S6 File, in which we also include graphs for all our results with the response variable ***S***_***max***_***/N***, i.e. normalized size of largest region, as used by Flache et al [21].

Qualitatively, the replications exhibited an equivalent behavior to the originals, especially regarding stability against noise. Statistically, we do not find a significant difference between the model implementations of Axelrod's model, with F(1, 1176) = 0.007, p = 0.935. However, we find a significant difference between the model implementations of Flache's model, with F(1, 1176) = 80.491, p < 0.0001, as our code resulted in slightly higher levels of diversity in our implementation of Flache's model, compared to their original results. However the effect size was found to be small (ηp^2^ = 0.064).

**Fig 4:**
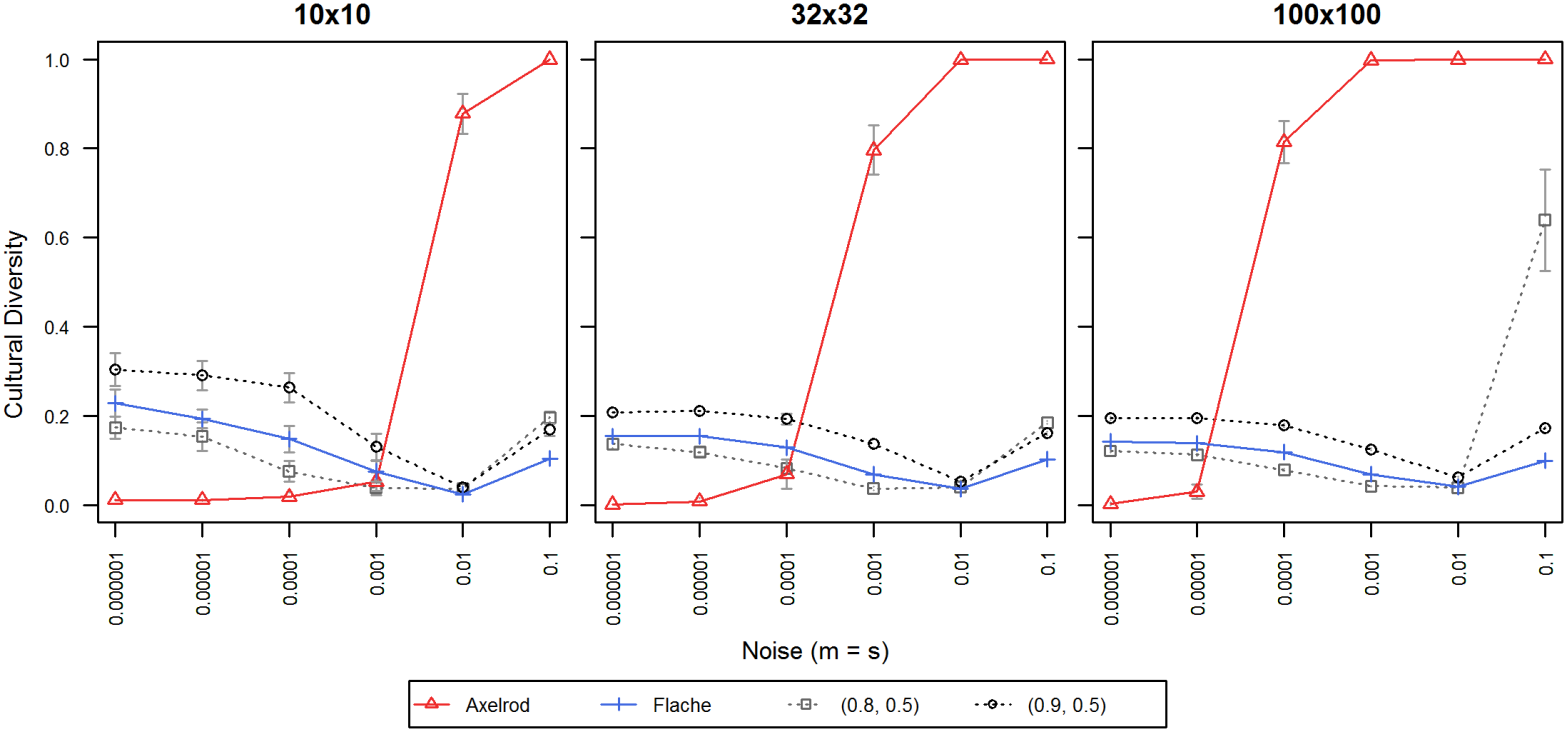
Cultural diversity for different models (Axelrod's, Flache's, and ours). X-axis displays levels of noise; Y-axis displays normalized cultural diversity. Each colored line symbol denotes one of three models, i.e. Axelrod's (continuous red), Flache's (continuous blue), ours with α = 0.9 and α' = 0.5 (dotted black) and ours with α = 0.8 and α' = 0.5 (dotted gray). 95% confidence intervals are displayed only when exceeding the size of the line symbol. Data points are averages of 50 repetitions per territory with 100,000 iterations per agent.

From experiment A, we chose institutional influence ***α*** = 0.8 and 0.9 f or the graphs presented in Fig 4, and compared them with our implementation of Axelrod's and Flache's models at various levels of noise, with our values otherwise equivalent to Fig 3.

Consistent with previous research [21,23,24], in our replication, Axelrod's model was highly sensitive to noise (and the threshold where monoculture turns to anomie decreased for bigger populations), while Flache's did not display this high sensitivity to noise, just as in the original research [21]. Our model exhibited a similar robustness at higher studied levels of institutional influence, ***α*** > = 0.8 (except in model 100x100/0.8/0.5 and noise level at 0.1).

In terms of the number of cultural regions, while for ***α*** < = 0.8, our diversity generally fell below the levels researched by Flache's model, at a high institutional influence ***α*** > = 0.9, our model was able to sustain more diversity than Flache's across all levels of noise and population sizes, as is evident from Fig 4.

#### Experiment C: Agent loyalty

**Fig 5:**
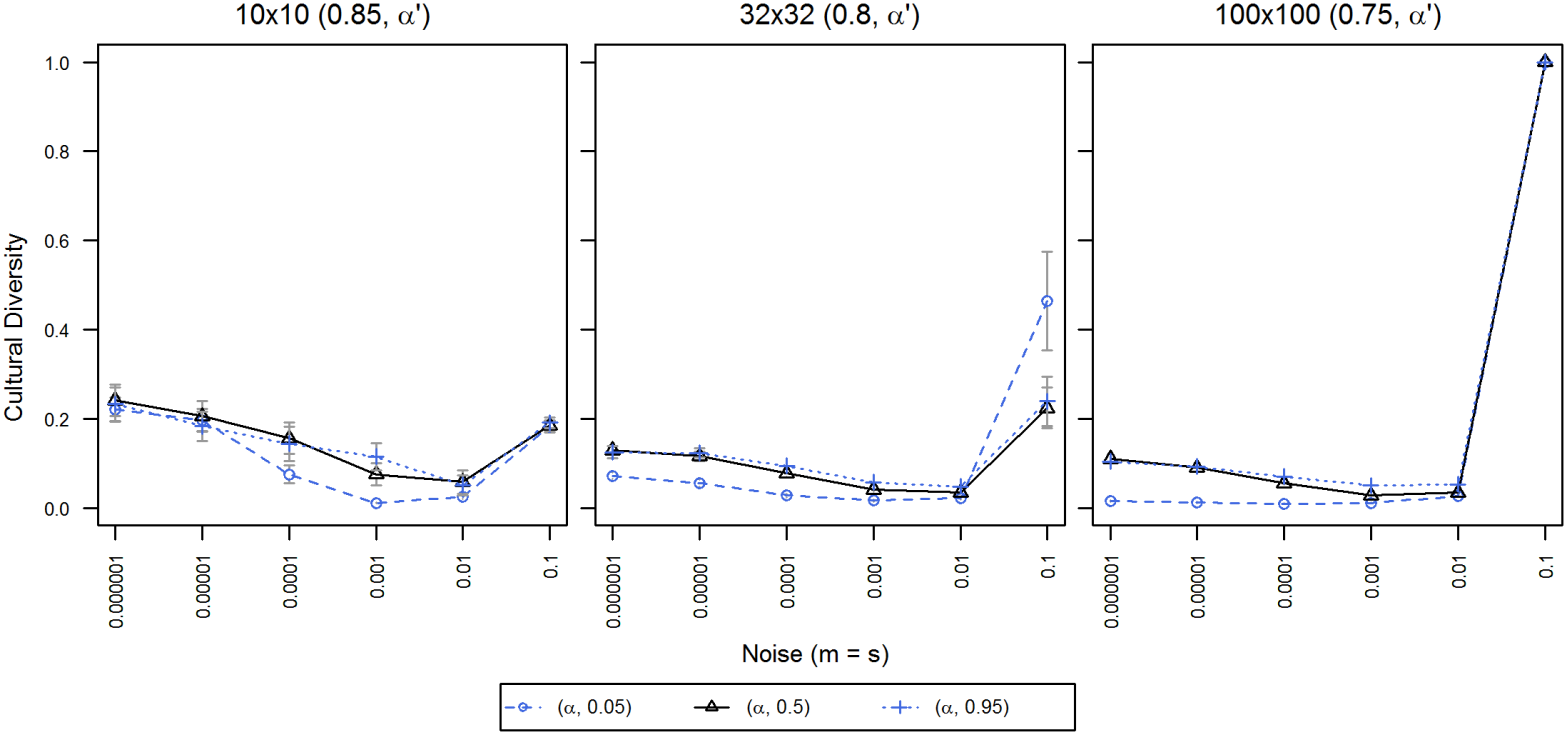
Cultural diversity for different levels of agent loyalty. X-axis displays levels of noise; Y-axis displays normalized cultural diversity. Each line symbol denotes one alpha prime of agent loyalty. 95% confidence intervals are displayed only when exceeding the size of the line symbol. Data points are averages of 50 repetitions per territory with 100,000 iterations per agent.

For experiment C, we chose three different values of institutional influence: ***α*** = 0.85 for the population of 10x10, ***α*** = 0.8 for 32x32, and ***α*** = 0.75 for 100x100. Fig 5 illustrates results obtained by manipulation of agent loyalty (***α'***) in addition to the just mentioned **α** values that we chose as institutional influence.

Unlike in the previous figures, a visual analysis of the graphs does not provide a clear overview over the effects, however, a statistical analysis yields some information: across all populations, low values of loyalty (***α'*** = 0.05) (compared to the baseline (***α'*** = 0.5)) did significantly reduce the level of diversity, F(1, 1764) = 32.57, p < 0.0001. This is particularly visible in Fig 5 for the medium and large populations and noise < = 0.0001. An analysis of high values of loyalty (α' = 0.95) produced an effect in the other direction, i.e. an increase of diversity, however, we only found significate differences for 32x32/0.8 and noise < = 0.01, F(1, 490) = 12.31, p = 0.00049; and for 100x100/0.75, F(1, 588) = 109.87, p < 0.0001. In general, effects were stronger for bigger populations and lower institutional influences. Detailed explanations of the calculated F-values and significance levels can be found in S2 File.

#### Discussion Experiments A to C

The first three experiments extend on previous institutional research [26–36] by directly modelling the effects of institutions on social influence processes and diversity. Our primary findings establish that institutional influence is successful in preserving cultural diversity by allowing multiple cultural regions to exist simultaneously in a stable system. Agents' loyalty to their institutions did play a small role in the preservation of diversity, however, it did not facilitate any increases. In multiple cases, our experiments compare favorably with previous results obtained by Axelrod [5] and Flache et al [21] in terms of stability and diversity.

Extending on the main result obtained in experiment A, we found that various amounts of institutional influence (***α*** > = 0.7) impact the extent of diversity that can be obtained and can be used to control the number of cultural regions that will emerge. It is important to consider the implications of the values that are presented in our model: an institutional influence ***α*** < 0.5 reflects that agents are giving more importance to their neighbor's opinion than to that of their institution. Probabilistically speaking, if an institutional influence value of α < 0.5 privileges the neighbor's influence, it is unsurprising that our results converge towards results obtained in Axelrod's original model, in which the neighbor's influence (regulated by homophily) is the only factor that matters. Notice that our model with ***α*** = 0 implies removing homophily from Axelrod's model, a scenario where the neighbor's influence is extreme. Thus, values around ***α*** = 0.7 are not in reality as high as they might first appear.

We found adequate and stable results for values of ***α*** between 0.7 and 0.95, and experiment B clarifies that our model with high levels of institutional influence (***α*** > = 0.9) was able to sustain even more diversity than Flache's model of multilateral social influence [21], which, to our knowledge, had yielded the best results so far in terms of diversity and stability. Additionally, for high levels of institutional influence, our model proved to be resilient when tested against the same levels of noise as Flache's model.

We did not find any strong effects in experiment C, when analysing the impact on agent loyalty, although a small impact on preserving diversity was established. The main obstacle in this case seems to be that ***α'*** is applied as a factor only in a very limited number of occasions, as it heavily depends on the initial institutional influence, i.e. the institution has to allow a trait change in the first place before an agent gets to decide whether they will switch to another institution (see Rule 3.1 and Rule 3.2 in Table 2), thus reducing the probability of institutional change. We found that the model in which we used the lowest alpha (100x100/0.75/0.5 in experiment C) showed the strongest effect of agent loyalty, which provides some empirical support for this post-hoc hypothesis. The alternative hypothesis, i.e. that the size of the population is the explanatory factor, is not supported by Experiment A, as our data shows that population size had no strong effects in any of the simulations. However, experiments that specifically address this hypothesis will be necessary to expand on our findings.

We found that, in our models, the normalized number of cultural regions was proportional to the population (when ***α***/***α'*** is held constant) which suggests that our results are scalable, i.e. the results hold regardless the population size. Consequently, there is a linear trend for the absolute number of cultural regions, where the bigger the population, the more cultures emerge. This implies that the size of the cultural regions (i.e. number of agents per cultural region on average) is similar across populations, but changes for each model through the given ***α*** and ***α'*** values. Thus, an alternative interpretation of our results is that the addition of strong institutions to the simulation impacted the size, not the number, of the cultural regions that emerged in the system. Our model here replicates the reversal behaviour of Axelrod's previous finding (that number of cultures decreases with increasing population size, p.219 in [5]), which had previously also been addressed and discussed by Flache et al (p.984 in [21]).

Lastly, our results in Table 4 show that, generally speaking, more cultures than institutions emerged in our models. This means that even under the influence of one institution, multiple agents can all belong to different cultures and those cultures can survive. In other words, an institution can allow the simultaneous existence of several cultures. This result is consistent with Shibanai et al [18], in which mass media, as a globally acting entity, was also found to promote the emergence of cultural diversity.

So far, our models support the idea that institutional influence can be used to control cultural diversity. In the following experiments D to F, we will now extend our study of institutions to include two mechanisms of influence used by and on institutions: democracy and propaganda.

### Section 2: Experiment D to F

In this second set of experiments, we show how two directions of institutional influence affect the system: bottom-up (democracy) and top-down (propaganda). We explore how they impact cultural regions and institution numbers when we apply the influence-loyalty model from experiment C, i.e. model values of 10x10/0.85/0.5, 32x32/0.8/0.5 and 100x100/0.75/0.5.

#### Experiment D: Rare and frequent democratic processes

In our democratic model, we manipulated the frequency at which democratic processes (referenda) occur in a system. We defined a unit of time as equivalent to the number of iterations necessary to have one iteration on average per agent, i.e. a unit of time is equivalent to 100 iterations in 10x10; 1024 in 32x32; and 10000 in 100x100. Then, a period is the duration of time between events. For example, a period of 10, i.e. a frequency of 1/10, means that the referenda occur after an average of 10 iterations per agent. We have set the frequency in exponential decrements of 1/10 (high democracy), 1/100 (moderate democracy) and 1/1000 (low democracy), in order to study a broad spectrum of possible values.

**Fig 6:**
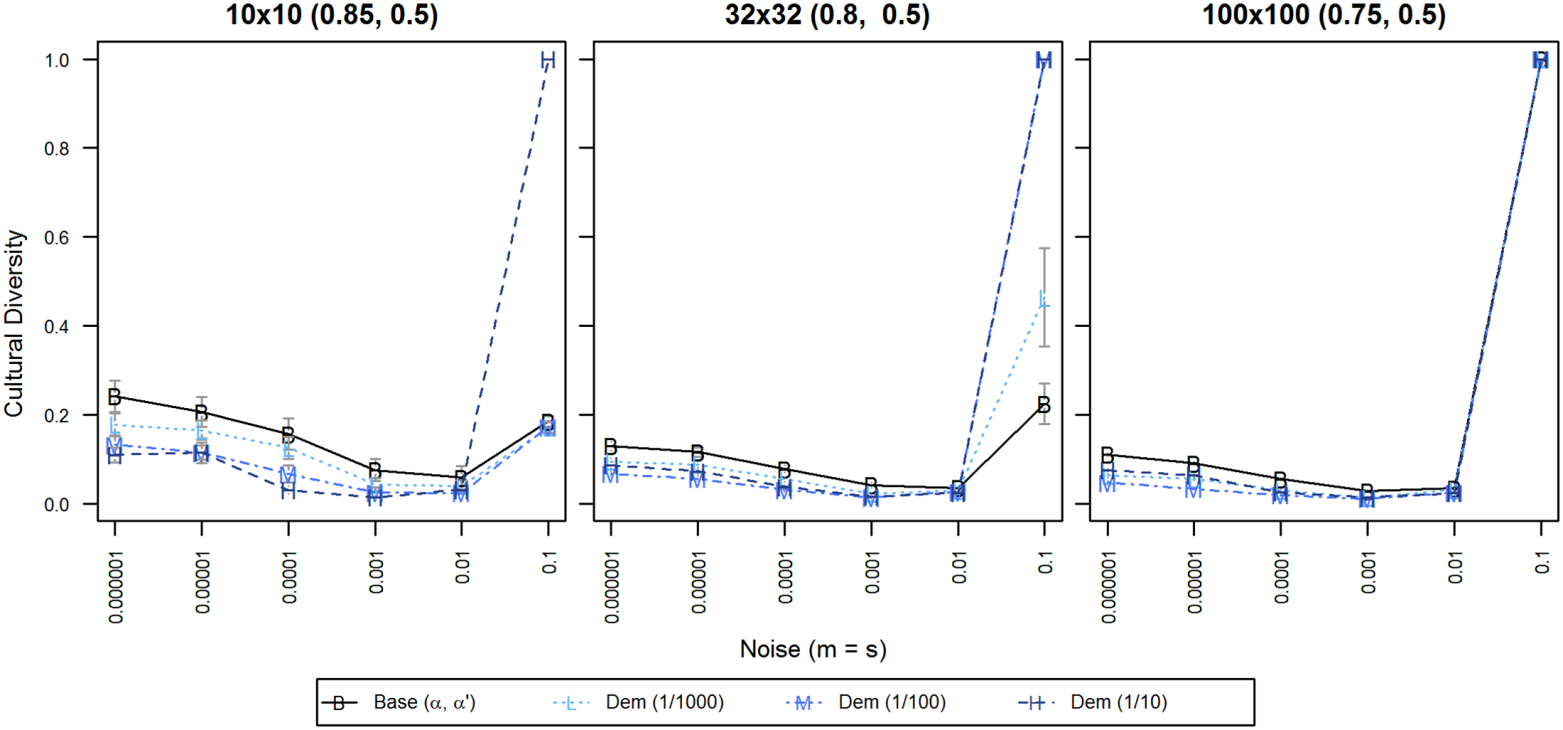
Cultural diversity for different frequencies of democracy. X-axis displays levels of noise; Y-axis displays normalized cultural diversity. Each colored line symbol denotes one frequency of democracy, from low (light blue) to high (dark blue). 95% confidence intervals are displayed only when exceeding the size of the line symbol. Data points are averages of 50 repetitions per territory with 100,000 iterations per agent.

As can be seen in Fig 6, in a model where democratic processes are allowed, cultural diversity was sustained, but it was strongly reduced compared to the previous baseline of diversity that we achieved in experiment A. This was the case at all three frequencies of democracy and all population sizes. Additionally, for the 10x10 population, the lower the democracy, the higher the diversity, when noise < = 0.01, with F(2, 735) = 48.806, p < 0.0001. For populations > = 32x32 this effect was non-monotonous. The lowest amount of diversity was reached by allowing moderate democracy (1/100). The difference was significant when compared to low democracy (1/1000) when noise < = 0.01, with F(1, 980) = 259.595, p < 0.0001, as well as compared to high democracy (1/10), with F(1,980) = 181.324, p < 0.0001 when noise < = 0.01. We present the results with noise < = 0.01 to avoid the extreme effect with noise = 0.1 observed in the figure, although that effect is consistent with our results. For further details on the performed ANOVA calculations, please refer to S3 File.

With regards to robustness, the system with added democracy turned somewhat unstable from a noise level of 0.01 onwards; at noise equals to 0.1, a state of anomie was reached for low democracy in 100x100, for medium democracy in 32x32 and 100x100, and for high democracy in all population sizes. Thus, an exploration of even more frequent democratic processes was deemed unnecessary; the results indicated that higher levels of democracy would only further destabilize our model against noise.

#### Experiment E: Rare and frequent propaganda processes

Just as with democracy in experiment D, in our model with propaganda, we manipulated the frequency at which propaganda processes occur in a system. When one looks at the occurrence of these two political tools, one finds that referenda are, in reality, rare (p.76 in [44]), whereas instances of propaganda are quite common and frequently encountered [45], so this time, we used higher frequencies of 1/1, 1/3 and 1/5. When we attempted rarer frequencies of propaganda in an earlier exploratory analysis, aligned with our predictions, once propaganda becomes too rare, effects become indiscernible.

**Fig 7:**
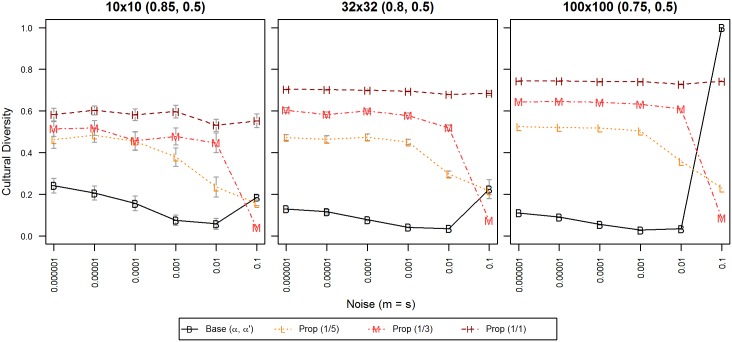
Cultural diversity for different frequencies of propaganda. X-axis displays levels of noise; Y-axis displays normalized cultural diversity. Each line symbol denotes one frequency of propaganda, from low (light red) to high (dark red). 95% confidence intervals are displayed only when exceeding the size of the line symbol. Data points are averages of 50 repetitions per territory with 100,000 iterations per agent.

As can be seen in Fig 7, our model with propaganda generated many co-existing cultural regions, i.e. in general, more propaganda led to more diversity. The only exception of this effect was found at the highest value of noise (0.1). At this level of noise, it was the rarer level of propaganda (1/5) which yielded more diversity than the moderate level (1/3).

In terms of resilience against noise, propaganda was able to stabilize the system even at the highest levels of noise (0.1). However, we found noticeable variations of diversity for the chosen frequencies of propaganda under higher levels of noise. For example, there was a tendency to monoculture, i.e. diversity was reduced significantly at noise levels = 0.01 and 0.1 and when propaganda was rare to medium frequent. This is a clear departure from the behavior of our previous models, where we so far tended to observe a convergence to anomie, which is better substantiated theoretically (as the highest possible noise value (1.0) can be equated with anomie).

#### Experiment F: Referendum + Propaganda

**Fig 8:**
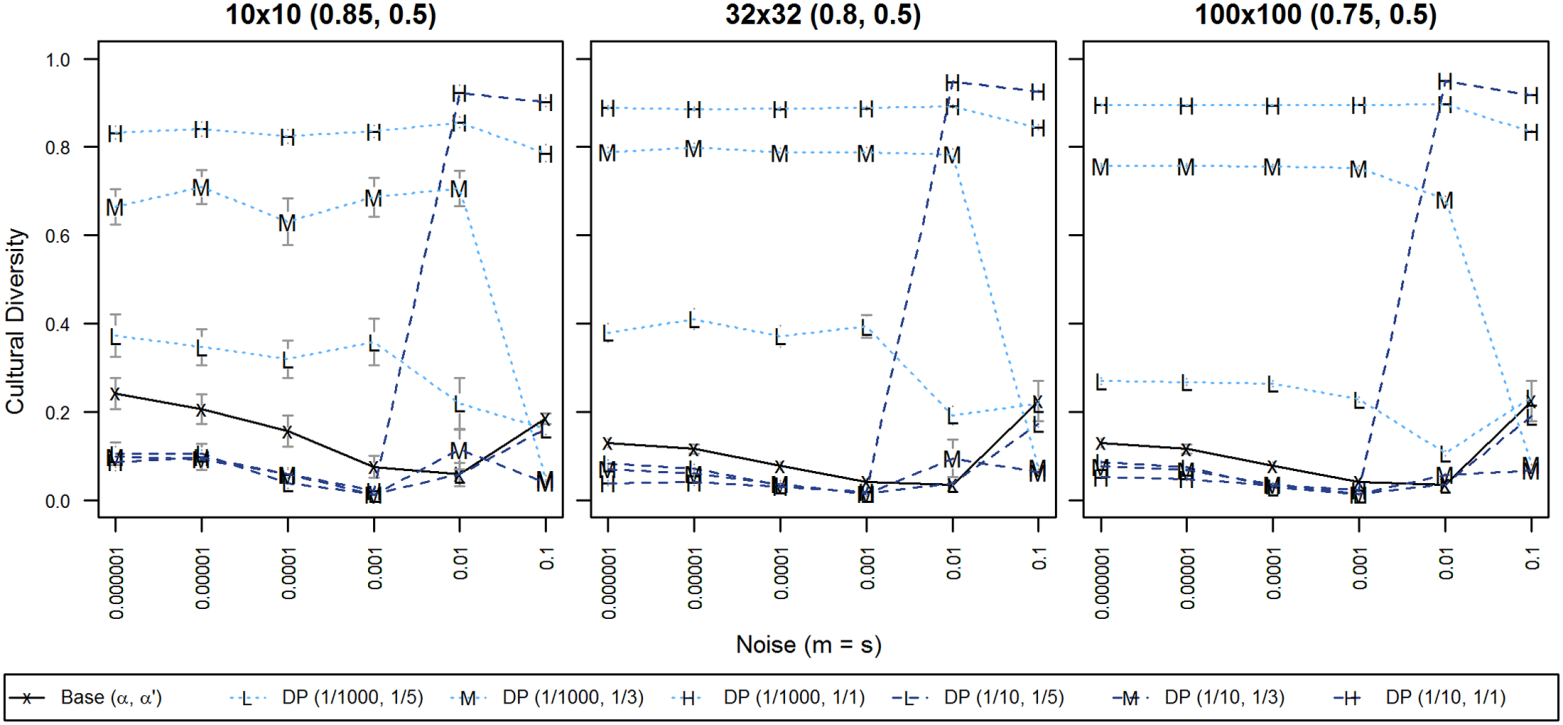
Cultural diversity for combinations of democracy and propaganda frequencies. X-axis displays levels of noise; Y-axis displays normalized cultural diversity. Each line symbol denotes one combination of democracy and propaganda. 95% confidence intervals are displayed only when exceeding the size of the line symbol. Data points are averages of 50 repetitions per territory with 100,000 iterations per agent.

Our final experiment explored what effects the combination of the two studied process in experiments D and E, democracy and propaganda, would have on the diversity in our system. We combined the two processes, generating a feedback loop of information that flows from individual to institutions (democracy) and vice versa (propaganda), so that institutional influence could run in both directions. This idea has been implicitly proposed in previous institutional research [35]. We manipulated the frequency at which both these processes occur in a system with the same values as we explored before, so for propaganda, we applied it on average every 1/1, 1/3 and 1/5 of interactions, while for democracy, we chose to apply it at frequency levels of 1/10, 1/100 and 1/1000; in the following graph (Fig 8), we omitted medium democracy (1/100) for brevity and readability, as it did not add additional information, i.e. it followed the prevailing trend described below. For the graph including medium democracy, please refer to S8 File.

In our feedback loop of institutional influence, the main effects of propaganda and democracy were partly confirmed from previous experiments: high levels of democracy still produced less diversity than baseline values that we obtained in experiment C (black lines in Fig 8), and also significantly less than low democracy, similar to experiment D. Furthermore, when we analyzed effects of propaganda interacting only with low democracy, propaganda held a positive relationship with diversity, i.e. the more propaganda, the more cultural regions, just as was the case in experiment E.

The effects are more difficult to discern for common democratic processes (1/10). For noise levels < = 0.001, differences in diversity are small, and for noise levels > = 0.01, the model turns very sensitive to propaganda. The interaction of democracy and propaganda for noise levels < = 0.00001, however, significantly impacts diversity. In this situation, the way propaganda impacts diversity was reversed, i.e. a system with high propaganda and high democracy produced less diversity, while less propaganda in a state of high democracy produced more cultural regions. This effect is only marginally visible in Fig 8, but statistically, we found a significant difference for populations > = 32x32, with F(2, 588) = 142.552, p < 0.0001. Further details regarding our calculations can be found under S4 File.

Finally, we would also like to present some data with regards to the numbers of institutions for this last experiment. Data and figures illustrating institutional numbers from experiments A, C, D and E can be found under S7 File.

**Fig 9:**
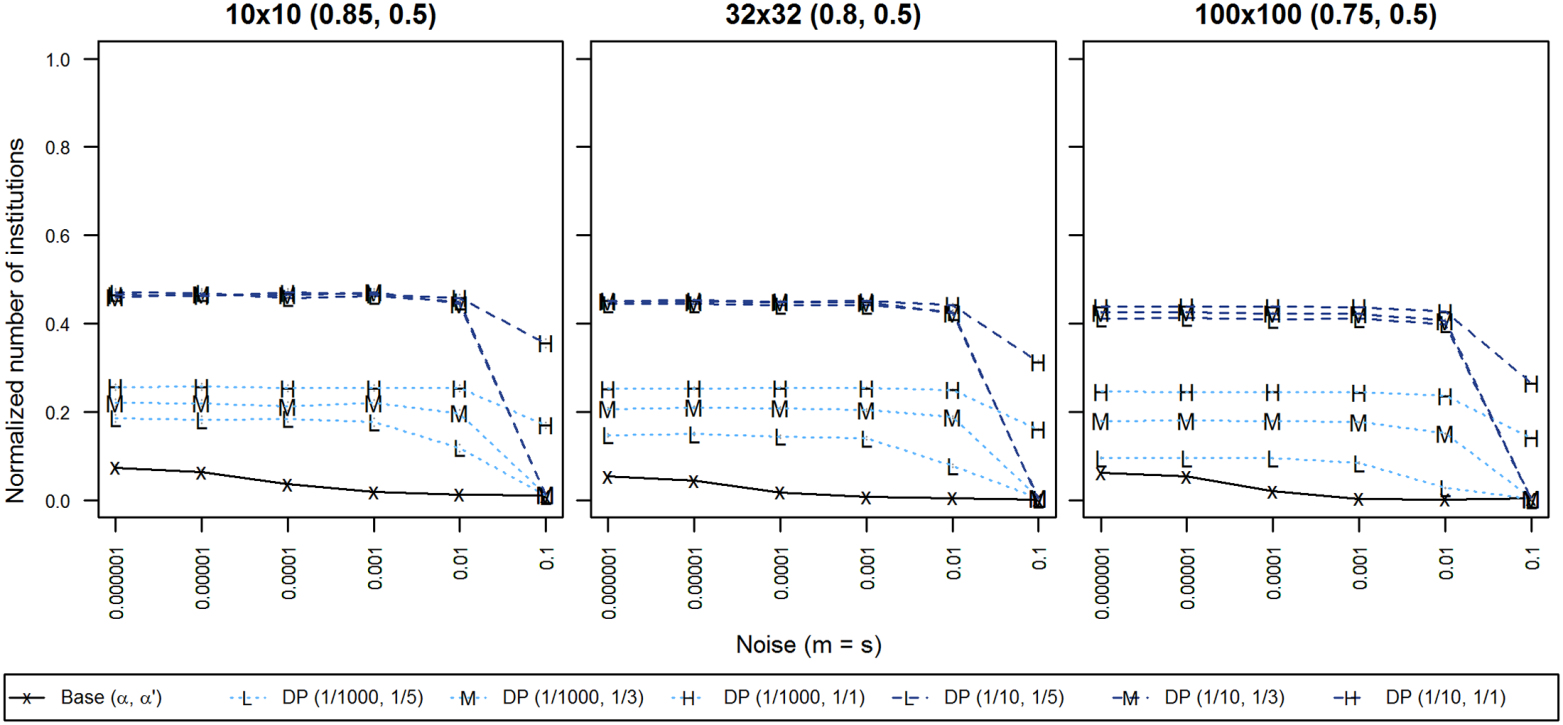
Number of institutions for combinations of democracy and propaganda frequencies. X-axis displays levels of noise; Y-axis displays normalized numbers of institutions. Each line symbol denotes one combination of democracy and propaganda. 95% confidence intervals are displayed only when exceeding the size of the line symbol. Data points are averages of 50 repetitions per territory with 100,000 iterations per agent.

We previously indicated in Table 4 that the number of institutions is generally smaller than the number of cultures across all our models. As illustrated in Fig 9, in this combined model, both democracy and propaganda increased the number of institutions when compared to the baseline. Democracy had the stronger effect. These effects were qualitatively similar for a separate analysis of democracy (Figure C in S7 File), and propaganda (Figure D in S7 File), but somewhat less extreme in their individual applications, i.e. numbers of institutions tended to be smaller than shown here. In particular for high levels of democracy, the number of institutions increased such, that it was higher than the resulting number of cultural regions, i.e. one cultural region could be governed by multiple different institutions.

#### Discussion Experiment D to F

Although the goal of democracy and propaganda is the same; i.e. to increase similarity between agents and institutions in order for each to exert more influence on the other, the impact of the two processes on cultural diversity was found to be opposite. Generally speaking, frequent democratic processes led to fewer, larger cultural regions (as shown in experiment D), while frequent propaganda led to more, smaller cultural regions (as shown in experiment E). Our results also reflect intuitive assumptions about these processes: propaganda needs to be common to succeed, so rarer frequencies did not produce any relevant results; referenda are rarer, and we found that the system destabilized more quickly when they were permitted too frequently.

When both institutional influence processes are combined, we found that the results observed in experiments D and E were reinforced, except in a state of high democracy, where higher frequencies of propaganda induced a more homogenous state in the population.

With regards to institutional numbers, we found that that they increased in the presence of democracy and propaganda. One possible explanation for this increase is that the feedback loop of institutional influence that exists in our model allows for a more consistent exchange of information between agents and institutions, so that the numbers of cultures and institutions converge more. Additionally, we found that frequent democratic processes increased the number of institutions even more; in fact, for models with high democracy we found that there were more institutions than cultures. This is the case when one culture is split into regions that each have its own institution, but are culturally identical. In this situation, a number of agents who belong to the same culture each subscribe to a different institution.

It is important to highlight that by using different combinations of propaganda and democracy, we are able to control most of the cultural diversity spectrum. Several combinations prove very successful at preserving diversity without destabilizing the system (i.e. not resulting in extremes such as anomie or global convergence), even when the noise is set at its highest level.

## General Discussion

Over the course of six experiments, we explored the effects of institutions on cultural diversity. We found that our model of institutional influence and agent loyalty compare well against previous models proposed by Axelrod [5] and Flache et al [21]: high levels of institutional influence successfully promoted diversity and sustained it against perturbations in our system, and the agents' loyalty helped preserve this diversity, though it did not further increase or impact it in any significant way.

The promotion of diversity can be understood if we look at the mechanism of institutional influence. The more an institution affects individuals' lives, the more it controls interactions between people, their traits and values, and how they socially influence each other. Strong institutions can keep people of one cultural belief system from associating with people from other cultures, which leads to isolation and reduces assimilation. This becomes particularly evident when we consider societies in which familial units play a very strong influencing institutional role: in cases where this is true, people are much less likely to interact about cultural beliefs with other "rival" families, or take on the cultural beliefs of strangers; instead, distrust towards the general public, political and social isolation and selfishness towards outgroups are found to be predominant [46,47].

Aside from simple institutional influence, the second goal of our research was to investigate how individuals' power to change their institutions affects the world in which they live, and what happens when institutions attempt to directly convince their members to re-adopt more traditional traits, even when those members might have been tempted away towards new beliefs. To study this, we implemented bottom-up and top-down institutional influence processes. Here, we found that democracy (bottom-up influence) promoted global convergence, whereas propaganda (top-down influence) by itself boosted diversity.

A possible explanation for this divergent result can be found in the source of institutional traits. In both cases, agents are the initiators of institutions. However, in the propaganda model, institutions are created, and then they preserve their configuration, they are fixed. "Old" traits are kept in the system, even when agents change towards more popular cultural opinion (due to interactions with neighbours). The traits that are stored in the institutions can be reused to influence agents once again through propaganda. This way, many small pockets of cultural regions can emerge and re-emerge.

Institutions' traits in the democracy model do not stay fixed. They are modified when agents are influenced by interactions with their neighbours, i.e. institutions are updated with the more recent, popular traits as old traits are abandoned by their agents. These new traits have successfully spread through the population and are converging agents' cultures. A bottom-up process generates the possibility that these new traits permeate the institutions as well, and institutions in turn can help to preserve these traits in the future. This way, less diverse, larger cultural regions emerge.

In our final model, in which we integrated both bottom-up and top-down influence in a feedback loop of information, we replicated the main effect of democracy, and the main effect of propaganda under low democracy. Additionally, we found an interaction in which the inclusion of high levels of democracy led to a reversal of propaganda effects: now, lower levels of propaganda increased diversity, while higher levels of propaganda reduced the amount of diversity in the system. Considering the previous explanations of the origins of institutional traits, we found a similar logic operating behind this interaction: if institutions can be modified towards the traits that are popularized across cultures (which then tend to convergence), the institutions' propaganda then does not reverse agents' traits back to 'older' values; they instead now help spread the new ideas that are growing popular in the population through propaganda, and if these propaganda processes are very frequent, they homogenize the population even more than before.

Measuring amounts of cultural diversity and frequencies of the mentioned institutional processes (such as how much democratic power people exert and how much propaganda exists) is very difficult in real societies. One attempt to apply our ideas can be to look at how cultural diversity is commonly perceived across the world. For example, we find a highly fragmented landscape with many small, diverse cultural pockets across the African continent (i.e. Chad alone holds around 100 distinct ethnic groups), which tends to also be low in democracy and high in propaganda, compared to for example a Western European political landscape which is more democratic, and arguably less diverse (i.e. we commonly use the term "Western culture" to describe many features that are identical across it) [48,49].

One concrete "trait" that can be mentioned, which is spreading across already fairly similar cultural regions through democracy and propaganda, and which is turning those regions more similar, is marriage equality; the idea of tolerance towards sexual orientations has been expanding across the Western world in recent years, with multiple referenda being held on the topic [50–52]. This movement, in turn, has been taken up by the media, is popularized further through positive institutional portrayals of tolerance (such as in school curriculums), and has successfully led to new, more inclusive laws in some countries. This stands in stark contrast to many smaller, autocratically governed areas across the world where homosexuality is treated very differently, ranging from ostracism over criminalization to punishment by death penalty [53]. This finding is reminiscent of Flache's hypothesis that maybe, ironically, conformist cultures are able to sustain more diversity than individualistic ones (p.990 in [21]), and that not all cases of persistent diversity are necessarily positive, as sometimes they can be a disguise for xenophobic and ostracizing, discriminative tendencies [21,54,55].

### Limitations and further research

We have substantially extended the current line of research on cultural diversity on a theoretical level by incorporating central authorities, i.e. institutions, for the first time, and by providing a system which, for future research, will facilitate controlling the full spectrum of possible diversity levels. However, three of our findings in particular will need to be clarified by further research.

Firstly, we only found a small effect of agent loyalty; it was able to preserve diversity but not increase it. We assume the main reason for the small size of the effect is that an agent's change of institutions is dependent on the probability of it changing its trait first. We added this assumption to the model because we perceived that realistically, it is unlikely that a person will change their institutional affiliation to that of their neighbor if the neighbor did not convince them of their cultural trait in the first place. Further research into the agent loyalty parameter when it is conceptualized as independent of institutional influence should clarify if it will indeed stay a small effect or have a bigger impact in its own right.

Secondly, we didn't find a clear relationship between the number of institutions and the number of cultural regions (Experiment F, Fig 9); in some cases they were more cultural regions than institutions, but in other cases, the reverse was true. In real life settings, both options are possible: one cultural region can be governed by multiple institutions, and one institution govern multiple cultures, however, we cannot be sure how this impacts diversity. From our results, we hypothesize that artificially manipulating the number of institutions would not consistently change the resulting diversity (in either direction), but this should be clarified in further studies.

Thirdly, the question remains how cultural diversity can be sustained even when institutions are permeated by novel ideas that are gaining approval in the population (i.e. under democracy). We hypothesize that democratic institutions still exert enough influence to slow down cultural drift patterns that would otherwise lead to complete monoculture. Possibly, allowing influences from other cultural regions to permeate institutions is what promotes the here established levels of cultural diversity. Further research should consider not only investigating the amount of cultural diversity that exists in a system, but also use a measure of frequency at which cultural change has occurred inside those cultural regions.

We also consider important the integration of multilateral social influence into our model of institutions, which has been used previously to induce and maintain cultural diversity [20,21]. In this sense, further research can also consider the inclusion of new parameters that expands the conditions in which the interactions occur, for example the distinction between normative and informational social influence [56], agents' differing personalities (openness, desire for control [57,58]), situational factors (such as cultural resilience in the presence of peace and war, wealth and poverty [59,60]) or the possibility of agent mobility within the system [15,16].

Finally, we found that little research has investigated the patterns presented here in real life settings as of yet. Field studies and experimental research on the impacts of institutions on diversity need to be carried out in order to test the practical and empirical relevance of our model's predictions.

## Supporting Information

S1 File Diversity differences by populations. (PDF)

S2 File Diversity differences by agent loyalty. (PDF)

S3 File Diversity differences by democracy. (PDF)

S4 File Diversity differences by democracy/propaganda combined. (PDF)

S5 File Stable states of equilibrium. (PDF)

S6 File Replication of Axelrod/Flache's results. (PDF)

S7 File Number of institutions. (PDF)

S8 File Complete results Experiment F (inclusion of democracy 1/100). (PDF)
