# Supplementary material for: Institutions and Cultural Diversity: Effects of Democratic and Propaganda Processes on Local Convergence and Global Diversity
Source: PLoS One. 2016 Apr 8;11(4):e0153334. doi: 10.1371/journal.pone.0153334 (PMC4825973; doi:10.1371/journal.pone.0153334)
Supplement: S1 File — (PDF) [file pone.0153334.s001.pdf]

S1 File. Diversity differences by populations.

Legend:

Yellow: reported values

Green: main effects and interactions that corroborate reported results

Blue: means and standard deviations that drive the significant differences

Purple: alternate possible result that could have been reported

Noise = level of mutation (ranges from 0.000001 to 0.1)

Size = population sizes (10x10, 32x32, 100x100)

Alpha = level of institutional influence (usually between 0.5 and 0.95)

Alpha_prime = level of agent loyalty (values of 0.05, 0.5 or 0.95)

Diversity differences by populations

Although some differences are obvious in the Fig 3. of the main document, we found that the results seem fairly proportional, especially for the two biggest population (>=32x32) and higher alphas. Here is some of the evidence:

1. We found no statistically difference for alpha 0.95 as it is shown in **Table** 1, in which case not even the interaction with noise was significant.
2. For populations >= 32x32, if we just control for alpha >= 0.7, we find a significant difference for the population size with a considerable high F value in the main effect and interactions (see ANOVA 2 in **Table** 2). However, when we check the averages and standard deviations, it seems that two treatments are driving this main effect: 32x32/0.7/0.5 vs 100x100/0.7/0.5 and 32x32/0.8/0.5, 100x100/0.8/0.5 both with noise of 0.1 (see the averages highlighted in cyan in the averages of the **Table** 2). In order to statistically corroborate this we tested the following
    2.1. For populations >= 32x32, when we control for alpha >= 0.7 and noise <= 0.01, we didn't find any significant difference for the main effect of the population size (see ANOVA 2 in **Table** 2). The interactions did present a significant difference (highlighted green in ANOVA 2 in **Table** 2) although the low F values suggest a very small effect.
    2.2. For populations >= 32x32, when we control for alpha >= 0.9, we didn't find any significant difference for the main effect of the population size (see ANOVA 3 in **Table** 2). The interactions did present a significant difference (highlighted green in ANOVA 3 in **Table** 2) although the low F values suggest a very small effect.

For alpha = 0.95

Table 1 – Two-way ANOVA comparing main effect of populations on cultural diversity for alpha = 0.95.

```
Anova Table (Type I tests)
Response variable: Cultural Diversity

Factors: Noise*Size for alpha = 0.95
             Df Sum Sq Mean Sq F value               Pr(>F)
Noise         5  8.930  1.7859 332.844 <0.0000000000000002 ***
Size          2  0.002  0.0009   0.167                0.846
Noise:Size   10  0.073  0.0073   1.358                0.195
Residuals   882  4.732  0.0054
---
Signif. codes:  0 '***' 0.001 '**' 0.01 '*' 0.05 '.' 0.1 ' ' 1

Averages of the compared groups
     0.000001   0.00001    0.0001     0.001     0.01       0.1
10  0.3482000 0.3786000 0.3502000 0.3368000 0.109400 0.1608000
32  0.3586719 0.3646094 0.3567773 0.2993945 0.118125 0.1665625
100 0.3647700 0.3610040 0.3567580 0.2985580 0.123676 0.1744460

Standard deviations of the compared groups
       0.000001     0.00001      0.0001       0.001         0.01          0.1
```

```
10  0.12310092 0.14159456 0.15516272 0.14299194 0.085509517 0.041641816
32  0.03890176 0.03069054 0.04192173 0.03812455 0.021656675 0.028318704
100 0.01397752 0.01396468 0.01198097 0.01415235 0.007738047 0.008887763
```

For alpha > 0.7

Table 2 – Three-way ANOVA comparing main effect of populations on cultural diversity, ANOVA 1 including all noise and size models with alpha >= 0.7. ANOVA 2 subsets data by noise <= 0.01 for alpha >= 0.7, and ANOVA 3 displays results for all noises and sized, but for alpha >=0.9.

```
Anova Table (Type I tests)
Response variable: Cultural Diversity


ANOVA 1
Factors: Alpha(>=0.7)*Noise*Size
                    Df Sum Sq Mean Sq F value                Pr(>F)
Alpha                3   7.35   2.451  622.40 < 0.0000000000000002 ***
Noise                5  32.83   6.567 1667.44 < 0.0000000000000002 ***
Size                 1   0.21   0.209   52.99    0.000000000000454 ***
Alpha:Noise         15  58.87   3.925  996.57 < 0.0000000000000002 ***
Alpha:Size           3   0.59   0.195   49.57 < 0.0000000000000002 ***
Noise:Size           5   1.21   0.242   61.51 < 0.0000000000000002 ***
Alpha:Noise:Size    15   3.20   0.213   54.20 < 0.0000000000000002 ***
Residuals         2352   9.26   0.004
---
Signif. codes:  0 '***' 0.001 '**' 0.01 '*' 0.05 '.' 0.1 ' ' 1

ANOVA 2
Factors: Alpha(>=0.7)*Noise(<=0.01)*Size
                    Df Sum Sq Mean Sq  F value                Pr(>F)
Alpha                3 18.054   6.018 9207.907 < 0.0000000000000002 ***
Noise                4  4.989   1.247 1908.434 < 0.0000000000000002 ***
Size                 1  0.001   0.001    1.406               0.2359
Alpha:Noise         12  2.016   0.168  257.089 < 0.0000000000000002 ***
Alpha:Size           3  0.020   0.007   10.023           0.00000149 ***
Noise:Size           4  0.011   0.003    4.036               0.0029 **
Alpha:Noise:Size    12  0.011   0.001    1.350               0.1836
Residuals         1960  1.281   0.001
---
Signif. codes:  0 '***' 0.001 '**' 0.01 '*' 0.05 '.' 0.1 ' ' 1


ANOVA 3
Factors: Alpha(>=0.9)*Noise*Size
                    Df Sum Sq Mean Sq  F value                Pr(>F)
Alpha                1  4.399   4.399 7735.524 < 0.0000000000000002 ***
Noise                5  6.023   1.205 2118.309 < 0.0000000000000002 ***
Size                 1  0.001   0.001    1.638              0.20085
Alpha:Noise          5  1.243   0.249  437.001 < 0.0000000000000002 ***
Alpha:Size           1  0.005   0.005    9.636              0.00195 **
Noise:Size           5  0.018   0.004    6.256           0.00000975 ***
Alpha:Noise:Size     5  0.006   0.001    2.236              0.04861 *
Residuals         1176  0.669   0.001
---s
Signif. codes:  0 '***' 0.001 '**' 0.01 '*' 0.05 '.' 0.1 ' ' 1)


Averages of the compared groups
32x32:

      0.000001    0.00001     0.0001      0.001       0.01       0.1
```

0.7	0.1036328	0.07275391	0.04289063	0.01794922	0.02898437	0.9999609
0.8	0.1366016	0.11832031	0.08208984	0.03654297	0.03953125	0.1848047
0.9	0.2079883	0.21148437	0.19265625	0.13765625	0.05285156	0.1605859
0.95	0.3586719	0.36460937	0.35677734	0.29939453	0.11812500	0.1665625

100x100:

	0.000001	0.00001	0.0001	0.001	0.01	0.1
0.7	0.107312	0.079202	0.046138	0.024656	0.044248	0.999938
0.8	0.121038	0.112404	0.078220	0.041880	0.038652	0.639134
0.9	0.194498	0.194736	0.178012	0.124836	0.061804	0.173120
0.95	0.364770	0.361004	0.356758	0.298558	0.123676	0.174446

Standard deviations of the compared groups

32x32:

	0.000001	0.00001	0.0001	0.001	0.01	0.1
0.7	0.05196727	0.05134128	0.04172406	0.01776962	0.02527264	0.0001933087
0.8	0.03242833	0.03695716	0.03067827	0.01333619	0.01679011	0.0126984702
0.9	0.03494106	0.03223964	0.04148088	0.03021904	0.01137599	0.0134539850
0.95	0.03890176	0.03069054	0.04192173	0.03812455	0.02165668	0.0283187043

100x100:

	0.000001	0.00001	0.0001	0.001	0.01	0.1
0.7	0.01858153	0.012621095	0.014854215	0.010980877	0.024396071	0.00006667007
0.8	0.01066135	0.010140582	0.009223661	0.004281617	0.004210300	0.40198894726
0.9	0.01389960	0.009546316	0.012931636	0.009466024	0.004440397	0.00853676324
0.95	0.01397752	0.013964678	0.011980973	0.014152350	0.007738047	0.00888776297
