# Supplementary material for: Institutions and Cultural Diversity: Effects of Democratic and Propaganda Processes on Local Convergence and Global Diversity
Source: PLoS One. 2016 Apr 8;11(4):e0153334. doi: 10.1371/journal.pone.0153334 (PMC4825973; doi:10.1371/journal.pone.0153334)
Supplement: S2 File — (PDF) [file pone.0153334.s002.pdf]

# S2 File. Diversity differences by agent loyalty.

## Legend:

Yellow: reported values

Green: main effects and interactions that corroborate reported results

Blue: means and standard deviations that drive the significant differences

Purple: alternate possible result that could have been reported

**Noise** = level of mutation (ranges from 0.000001 to 0.1)

**Size** = population sizes (10x10, 32x32, 100x100)

**Alpha** = level of institutional influence (usually between 0.5 and 0.95)

**Alpha_prime** = level of agent loyalty (values of 0.05, 0.5 or 0.95)

# Diversity differences by agent loyalty

## For alpha_prime = 0.05 vs 0.5

We find a statistically difference for alpha_prime when we compare 0.05 vs 0.5 in the main effects and in the interactions. The low values of F suggest that the effects are small, which can be appreciated in Fig 5.

**Table 1 – Three-way ANOVA comparing main effect of alpha_prime on cultural diversity when alpha_prime 0.05 and 0.5.**

```
Anova Table (Type I tests)
Response variable: Cultural Diversity

Factors: Noise*Size*Alpha_Prime
                   Df Sum Sq Mean Sq  F value              Pr(>F)
Noise               5  49.66   9.931 1318.90 < 0.0000000000000002 ***
Size                2   2.69   1.347  178.92 < 0.0000000000000002 ***
Alpha_Prime         1   0.25   0.245   32.57         0.0000000135 ***
Noise:Size         10  37.94   3.794  503.89 < 0.0000000000000002 ***
Noise:Alpha_Prime   5   1.03   0.207   27.47 < 0.0000000000000002 ***
Size:Alpha_Prime    2   0.19   0.095   12.65         0.0000034979 ***
Noise:Size:Alpha_Prime  10   0.97   0.097   12.84 < 0.0000000000000002 ***
Residuals        1764  13.28   0.008
---
Signif. codes:  0 '***' 0.001 '**' 0.01 '*' 0.05 '.' 0.1 ' ' 1


Averages of the compared groups
10x10:
     0.000001 0.00001 0.0001  0.001   0.01    0.1
0.05   0.2204   0.1966 0.0754 0.0112 0.0244 0.1824
0.5    0.2416   0.2058 0.1558 0.0752 0.0582 0.1844

32x32:
       0.000001    0.00001     0.0001      0.001       0.01        0.1
0.05 0.07212891 0.05546875 0.02761719 0.01611328 0.02095703 0.4638867
0.5  0.12921875 0.11574219 0.07742187 0.04136719 0.03480469 0.2240820

100x100:
     0.000001  0.00001   0.0001    0.001     0.01      0.1
0.05 0.015178 0.012350 0.009108 0.011186 0.026418 0.999938
0.5  0.110166 0.090392 0.054666 0.027842 0.034284 0.999958


Standard deviations of the compared groups
10x10:
       0.000001    0.00001    0.0001       0.001        0.01        0.1
0.05 0.09774937 0.08913668 0.0723994 0.003282607 0.01342553 0.04569464
0.5  0.12253046 0.11770059 0.1249504 0.087975878 0.09272936 0.04248217

32x32:
       0.000001    0.00001     0.0001      0.001        0.01       0.1
0.05 0.02715806 0.02796937 0.01348416 0.00752120 0.009566712 0.3889801
0.5  0.03407586 0.03706017 0.02921841 0.01934477 0.012353845 0.1607923

100x100:
        0.000001     0.00001      0.0001       0.001        0.01            0.1
0.05 0.007169587 0.004478714 0.002681824 0.004027407 0.003808567 0.00008302938
0.5  0.010767517 0.012383314 0.008317589 0.005293126 0.006277636 0.00007024738
```

## For alpha_prime = 0.5 vs 0.95

In the case of the comparison between alpha_prime of 0.05 and 0.5, the differences become even smaller. First, we found and almost not significant effect for alpha_prime in ANOVA 1 (

1. **Table** 2). However,

When we control by population size = 32x32 and noise <=0.01 (ANOVA 2 in

2. **Table** 2), we find a significant difference. The low F value also suggest a very small effect.

When we control by population size = 100x100 (ANOVA 2 in

3. **Table** 2), we find a significant difference. In this case the F value is considerable, and the interaction with noise is also significant.

We see a higher significant for higher population sizes. However, we are using different values of alpha for different population sizes: 0.85 for 10x10, 0.8 for 32x32 and 0.75 for 100x100. It is more likely that the difference has to be attributed to the alpha values than the population size because:

1. We show in Experiment A that the results were fairly proportional across populations.
2. Alpha_prime is a dependant variable of alpha. We can appreciate this in Table 2, Step 3.

**Table 2 – Three-way ANOVA comparing main effect of alpha_prime on cultural diversity, with ANOVA 1 displaying results when alpha_prime 0.5 and 0.95. ANOVA 2 subsets data for noise levels <0.1 and population size 32x32 only. ANOVA 3 displays results for all noise levels and population size 100x100.**

```
Anova Table (Type I tests)
Response variable: Cultural Diversity


ANOVA 1
Factors: Noise*Size*Alpha_Prime
                          Df  Sum Sq Mean Sq  F value              Pr(>F)
Alpha_Prime                1    0.02   0.017    3.051              0.0809 .
Noise                      5   37.52   7.504 1311.228 <0.0000000000000002 ***
Size                       2    4.02   2.010  351.288 <0.0000000000000002 ***
Alpha_Prime:Noise          5    0.05   0.010    1.743              0.1215
Alpha_Prime:Size           2    0.01   0.006    0.981              0.3750
Noise:Size                10   39.84   3.984  696.190 <0.0000000000000002 ***
Alpha_Prime:Noise:Size    10    0.04   0.004    0.629              0.7901
Residuals               1764   10.10   0.006
---
Signif. codes:  0 '***' 0.001 '**' 0.01 '*' 0.05 '.' 0.1 ' ' 1

ANOVA 2
Factors: Noise(<0.1)*Alpha_Prime for Size=32x32
                    Df Sum Sq Mean Sq F value              Pr(>F)
Noise                4 0.6162 0.15405 157.902 < 0.0000000000000002 ***
Alpha_Prime          1 0.0120 0.01201  12.307            0.000493 ***
Noise:Alpha_Prime    4 0.0074 0.00186   1.908            0.107849
Residuals          490 0.4781 0.00098
---
Signif. codes:  0 '***' 0.001 '**' 0.01 '*' 0.05 '.' 0.1 ' ' 1


ANOVA 3
Factors: Noise*Alpha_Prime for Size=100x100
```

```
                    Df Sum Sq Mean Sq F value              Pr(>F)
Noise                5  72.63  14.527 141740.94 <0.0000000000000002 ***
Alpha_Prime          1   0.01   0.011    109.87 <0.0000000000000002 ***
Noise:Alpha_Prime    5   0.02   0.003     31.84 <0.0000000000000002 ***
Residuals          588   0.06   0.000
---
Signif. codes:  0 '***' 0.001 '**' 0.01 '*' 0.05 '.' 0.1 ' ' 1
```

**Averages of the compared groups**

**10x10:**

| | 0.000001 | 0.00001 | 0.0001 | 0.001 | 0.01 | 0.1 |
|---|---|---|---|---|---|---|
| 0.5 | 0.2416 | 0.2058 | 0.1558 | 0.0752 | 0.0582 | 0.1844 |
| 0.95 | 0.2326 | 0.1832 | 0.1436 | 0.1154 | 0.0506 | 0.1912 |

**32x32:**

| | 0.000001 | 0.00001 | 0.0001 | 0.001 | 0.01 | 0.1 |
|---|---|---|---|---|---|---|
| 0.5 | 0.1292188 | 0.1157422 | 0.07742187 | 0.04136719 | 0.03480469 | 0.2240820 |
| 0.95 | 0.1250000 | 0.1233789 | 0.09439453 | 0.05718750 | 0.04759766 | 0.2396094 |

**100X100:**

| | 0.000001 | 0.00001 | 0.0001 | 0.001 | 0.01 | 0.1 |
|---|---|---|---|---|---|---|
| 0.5 | 0.110166 | 0.090392 | 0.054666 | 0.027842 | 0.034284 | 0.999958 |
| 0.95 | 0.103798 | 0.093024 | 0.070262 | 0.049850 | 0.052394 | 0.999966 |

**Standard deviations of the compared groups**

**10x10:**

| | 0.000001 | 0.00001 | 0.0001 | 0.001 | 0.01 | 0.1 |
|---|---|---|---|---|---|---|
| 0.5 | 0.1225305 | 0.1177006 | 0.1249504 | 0.08797588 | 0.09272936 | 0.04248217 |
| 0.95 | 0.1312702 | 0.1166442 | 0.1340539 | 0.10725156 | 0.08092350 | 0.04288761 |

**32x32:**

| | 0.000001 | 0.00001 | 0.0001 | 0.001 | 0.01 | 0.1 |
|---|---|---|---|---|---|---|
| 0.5 | 0.03407586 | 0.03706017 | 0.02921841 | 0.01934477 | 0.01235384 | 0.1607923 |
| 0.95 | 0.04607260 | 0.03860573 | 0.03067649 | 0.03018372 | 0.01938804 | 0.1945799 |

**100X100:**

| | 0.000001 | 0.00001 | 0.0001 | 0.001 | 0.01 | 0.1 |
|---|---|---|---|---|---|---|
| 0.5 | 0.01076752 | 0.01238331 | 0.008317589 | 0.005293126 | 0.006277636 | 0.00007024738 |
| 0.95 | 0.01169407 | 0.01637560 | 0.015129912 | 0.009628047 | 0.009869566 | 0.00006262946 |
