# Supplementary material for: Institutions and Cultural Diversity: Effects of Democratic and Propaganda Processes on Local Convergence and Global Diversity
Source: PLoS One. 2016 Apr 8;11(4):e0153334. doi: 10.1371/journal.pone.0153334 (PMC4825973; doi:10.1371/journal.pone.0153334)
Supplement: S3 File — (PDF) [file pone.0153334.s003.pdf]

S3 File. Diversity differences by democracy.

Legend:

Yellow: reported values

Green: main effects and interactions that corroborate reported results

Blue: means and standard deviations that drive the significant differences

Purple: alternate possible result that could have been reported

Noise = level of mutation (ranges from 0.000001 to 0.1)

Size = population sizes (10x10, 32x32, 100x100)

Alpha = level of institutional influence (usually between 0.5 and 0.95)

Alpha_prime = level of agent loyalty (values of 0.05, 0.5 or 0.95)

Diversity differences by democracy

<u>For democracy = 1/10 vs 1/100 vs 1/1000, population 10x10</u>

For a population size of 10x10, we observe a monotonous result for democracy when noise <=0.01 in Fig 6. (main paper), i.e. the higher the democracy, the lower the diversity.

1. The ANOVA 1 in Table 1 shows a significant effect for democracy, although observing the Fig 6. it is clear that the noise = 0.1 is driving a big portion of the effect,

2. For noise < 0.1, ANOVA 2 in Table 1, democracy still shows a significant difference, and there is a significant effect for the interaction.

Table 1 – Two-way ANOVA comparing main effect of democracy on cultural diversity. First reported ANOVA displays results for population 10x10 only. ANOVA 2 displays subset results for noise <= 0.01.

```
Anova Tables (Type I tests)
Response variable: Cultural Diversity

ANOVA 1
Factors: Noise*Democracy for population size of 10x10:
                 Df  Sum Sq Mean Sq F value              Pr(>F)
Noise             5  18.547   3.709  1189.5 <0.0000000000000002 ***
Democracy         2   2.662   1.331   426.8 <0.0000000000000002 ***
Noise:Democracy  10  20.685   2.069   663.3 <0.0000000000000002 ***
Residuals       882   2.751   0.003
---
Signif. codes:  0 '***' 0.001 '**' 0.01 '*' 0.05 '.' 0.1 ' ' 1


ANOVA 2
Factors: Noise(<=0.01)*Democracy for population size of 10x10:
                 Df Sum Sq Mean Sq F value                Pr(>F)
Noise             4 1.7095  0.4274 122.464 < 0.0000000000000002 ***
Democracy         2 0.3406  0.1703  48.806 < 0.0000000000000002 ***
Noise:Democracy   8 0.1209  0.0151   4.331            0.0000407 ***
Residuals       735 2.5649  0.0035
---
Signif. codes:  0 '***' 0.001 '**' 0.01 '*' 0.05 '.' 0.1 ' ' 1

Averages of the compared groups
       0.000001 0.00001 0.0001  0.001   0.01    0.1
1/10     0.1094  0.1148 0.0302 0.0140 0.0306 1.0000
1/100    0.1318  0.1142 0.0664 0.0246 0.0234 0.1716
1/1000   0.1772  0.1650 0.1252 0.0426 0.0394 0.1712

Standard deviations of the compared groups
         0.000001     0.00001      0.0001       0.001        0.01         0.1
1/10   0.05950167 0.05406874 0.03100296 0.01511858 0.01391079 0.00000000
1/100  0.07678993 0.08119239 0.06598577 0.03796938 0.01334166 0.04210579
1/1000 0.08845707 0.07568059 0.08813811 0.05041906 0.04661654 0.04488716
```

For democracy = 1/10 vs 1/100 vs 1/1000, populations 32x32 and 100x100

For bigger populations sizes (or smaller alphas), the effect of democracy is non-monotonous. We observe the lowest values of diversity with moderate democracy (1/100). To corroborate this observation we decided to test the two contrast, Low (1/1000) vs Moderate (1/100) and Moderate (1/100) vs High (1/10):

1. For noises <= 0.01, the contrast 1/1000 vs 1/100 (ANOVA 3 in Table 1) shows a significant difference for democracy with a strong effect. The ANOVA 2 in Table 1 removes the control for noise which in the Fig 6. (main paper) is evident that is driving a strong effect; the significance persist, but as expected the effect is moved to the interactions.

2. For noises <= 0.01, the contrast 1/100 vs 1/10 (ANOVA 5 in Table 1) also shows a significant difference with a strong effect. The ANOVA 4 in Table 1 removes the control for noise which in the Fig 6. (main paper) is evident that is driving a strong effect; the significance persist, but as expected the effect is moved to the interactions.

A complete ANOVA removing the controls for noise and democracy is also shown for reference (ANOVA 1 in Table 1)

Table 2 – Three-way ANOVA comparing main effect of democracy on cultural diversity. ANOVA 1 displays results for population 32x32 and 100x100. ANOVAs 2 and 3 display subset results for democracy at 1/1000 and 1/100, and ANOVA 3 displays results for noise <= 0.01. ANOVAs 4 and 5 dusplay subset results for democracy at 1/10 and 1/100 and ANOVA 5 displays results for noise <= 0.01.

```
Anova Tables (Type I tests)
Response variable: Cultural Diversity

ANOVA 1
Factors: Noise*Size(>=32x32)*Democracy (1/1000,1/100, 1/10):
                       Df Sum Sq Mean Sq F value                Pr(>F)
Noise                   5 189.53   37.91 8675.34 < 0.0000000000000002 ***
Size                    1   0.17    0.17   38.17        0.000000000804 ***
Democracy               2   0.56    0.28   64.11 < 0.0000000000000002 ***
Noise:Size              5   2.32    0.46  106.01 < 0.0000000000000002 ***
Noise:Democracy        10   4.33    0.43   99.00 < 0.0000000000000002 ***
Size:Democracy          2   0.66    0.33   75.92 < 0.0000000000000002 ***
Noise:Size:Democracy   10   4.14    0.41   94.75 < 0.0000000000000002 ***
Residuals            1764   7.71    0.00
---
Signif. codes:  0 '***' 0.001 '**' 0.01 '*' 0.05 '.' 0.1 ' ' 1

ANOVA 2
Factors: Noise*Size(>=32x32)*Democracy(1/1000,1/100):
                       Df Sum Sq Mean Sq F value                Pr(>F)
Noise                   5 113.98  22.797 3513.33 < 0.0000000000000002 ***
Size                    1   0.30   0.304   46.84       0.0000000000123 ***
Democracy               1   0.31   0.305   47.01       0.0000000000114 ***
Noise:Size              5   3.38   0.676  104.11 < 0.0000000000000002 ***
Noise:Democracy         5   3.36   0.673  103.69 < 0.0000000000000002 ***
Size:Democracy          1   0.52   0.521   80.31 < 0.0000000000000002 ***
Noise:Size:Democracy    5   3.08   0.615   94.79 < 0.0000000000000002 ***
Residuals            1176   7.63   0.006
```

```
---
Signif. codes:  0 '***' 0.001 '**' 0.01 '*' 0.05 '.' 0.1 ' ' 1
```

ANOVA 3

Factors: Noise(<=0.01)*Size(>=32x32)*Democracy(1/1000,1/100):

```
Df Sum Sq Mean Sq F value                  Pr(>F)
Noise                  4 0.3808 0.09521 420.305 < 0.0000000000000002 ***
Size                   1 0.0594 0.05941 262.274 < 0.0000000000000002 ***
Democracy              1 0.0588 0.05880 259.595 < 0.0000000000000002 ***
Noise:Size             4 0.0292 0.00731  32.271 < 0.0000000000000002 ***
Noise:Democracy        4 0.0188 0.00470  20.750 < 0.0000000000000002 ***
Size:Democracy         1 0.0032 0.00321  14.187             0.000175 ***
Noise:Size:Democracy   4 0.0022 0.00056   2.452             0.044485 *
Residuals            980 0.2220 0.00023
---
Signif. codes:  0 '***' 0.001 '**' 0.01 '*' 0.05 '.' 0.1 ' ' 1
```

ANOVA 4

Factors: Noise*Size(>=32x32)*Democracy(1/100,1/10):

```
                       Df Sum Sq Mean Sq    F value                  Pr(>F)
Noise                   5 154.57  30.915 215284.136 < 0.0000000000000002 ***
Size                    1   0.02   0.019    129.615 < 0.0000000000000002 ***
Democracy               1   0.03   0.026    181.358 < 0.0000000000000002 ***
Noise:Size              5   0.01   0.003     18.730 < 0.0000000000000002 ***
Noise:Democracy         5   0.03   0.007     46.648 < 0.0000000000000002 ***
Size:Democracy          1   0.00   0.001      8.109              0.00448 **
Noise:Size:Democracy    5   0.00   0.000      2.684              0.02024 *
Residuals            1176   0.17   0.000
---
Signif. codes:  0 '***' 0.001 '**' 0.01 '*' 0.05 '.' 0.1 ' ' 1
```

ANOVA 5

Factors: Noise(<=0.01)*Size(>=32x32)*Democracy(1/100,1/10):

```
                      Df Sum Sq Mean Sq F value                Pr(>F)
Noise                  4 0.4327 0.10816 627.728 < 0.0000000000000002 ***
Size                   1 0.0224 0.02237 129.824 < 0.0000000000000002 ***
Democracy              1 0.0312 0.03124 181.324 < 0.0000000000000002 ***
Noise:Size             4 0.0097 0.00242  14.059     0.0000000000369 ***
Noise:Democracy        4 0.0283 0.00707  41.047 < 0.0000000000000002 ***
Size:Democracy         1 0.0014 0.00141   8.154             0.00439 **
Noise:Size:Democracy   4 0.0017 0.00042   2.446             0.04491 *
Residuals            980 0.1689 0.00017
---
Signif. codes:  0 '***' 0.001 '**' 0.01 '*' 0.05 '.' 0.1 ' ' 1
```

Averages of the compared groups

32x32:

	0.000001	0.00001	0.0001	0.001	0.01	0.1
1/10	0.08638672	0.07287109	0.03712891	0.01599609	0.02455078	0.9999414
1/100	0.06660156	0.05585938	0.03074219	0.01355469	0.02613281	0.9999023
1/1000	0.09386719	0.08761719	0.05460937	0.02238281	0.02902344	0.4639063

100x100:

	0.000001	0.00001	0.0001	0.001	0.01	0.1
1/10	0.074994	0.063752	0.025664	0.013628	0.023452	0.999946
1/100	0.046554	0.033660	0.018570	0.010562	0.024394	0.999972
1/1000	0.062926	0.055344	0.029582	0.013508	0.031136	0.999958

Standard deviations of the compared groups

32x32:

	0.000001	0.00001	0.0001	0.001	0.01	0.1
1/10	0.02478725	0.01947626	0.01302671	0.007270249	0.008854169	0.0002342747
1/100	0.01947936	0.02021195	0.01485654	0.012223785	0.017675908	0.0002959424

```
1/1000 0.02968469 0.02265209 0.02050149 0.011604996 0.017506053 0.3888418404

100x100:
         0.000001     0.00001      0.0001       0.001        0.01           0.1
1/10   0.00998407 0.010479294 0.005551163 0.004795620 0.003979039 0.00008621284
1/100  0.01294161 0.007113769 0.006142500 0.008590286 0.008499806 0.00005360475
1/1000 0.01259677 0.008950776 0.006065953 0.004614522 0.010260062 0.00006417451
```
