# Supplementary material for: Institutions and Cultural Diversity: Effects of Democratic and Propaganda Processes on Local Convergence and Global Diversity
Source: PLoS One. 2016 Apr 8;11(4):e0153334. doi: 10.1371/journal.pone.0153334 (PMC4825973; doi:10.1371/journal.pone.0153334)
Supplement: S4 File — (PDF) [file pone.0153334.s004.pdf]

S4 File. Diversity differences by democracy/propaganda combined.

Legend:

Yellow: reported values

Green: main effects and interactions that corroborate reported results

Blue: means and standard deviations that drive the significant differences

Purple: alternate possible result that could have been reported

Noise = level of mutation (ranges from 0.000001 to 0.1)

Size = population sizes (10x10, 32x32, 100x100)

Alpha = level of institutional influence (usually between 0.5 and 0.95)

Alpha_prime = level of agent loyalty (values of 0.05, 0.5 or 0.95)

Diversity differences by democracy/propaganda combined

<u>For democracy = 1/1000 and all propaganda levels = 1/5, 1/3, 1/1</u>

Apart from the sensitivity for propaganda after noise >= 0.01, other effects are difficult to perceive in Fig.8. This sensitivity is driving the main effect. For noise >= 0.001, we observe very little effects. However, we did find a significant difference (ANOVA 3 of **Table** 1), especially when we control for bigger population sizes (>=32x32), and high noises (>=0.0001). Although the effects are small, they are very interesting because they move in the opposite direction of Propaganda in Experiment E, when Democracy was not present; i.e. there is an interaction between propaganda and democracy.

Table 1 – Three-way ANOVA comparing main effect of propaganda on cultural diversity when democracy is rare (1/1000). ANOVA 1 displays results for all noises, propaganda frequencies, and populations. ANOVAs 2 and 3 displays results for subsetted data for noise levels below 0.0001. ANOVA 3 displays results only for populations 32x32 and 100x100.

```
Anova Tables (Type I tests)
Response variable: Cultural Diversity

ANOVA 1
Factors: Noise*Size*Propaganda(1/5,1/3,1/1):
                        Df Sum Sq Mean Sq   F value              Pr(>F)
Noise                    5  62.08  12.416  5951.435 < 0.0000000000000002 ***
Size                     2   0.09   0.046    22.182       0.000000000279 ***
Propaganda               2  44.89  22.446 10759.077 < 0.0000000000000002 ***
Noise:Size              10   0.25   0.025    11.881 < 0.0000000000000002 ***
Noise:Propaganda        10  96.86   9.686  4642.764 < 0.0000000000000002 ***
Size:Propaganda          4   0.01   0.002     0.783                0.536
Noise:Size:Propaganda   20   0.13   0.006     3.068       0.000005245431 ***
Residuals             2646   5.52   0.002
---
Signif. codes:  0 '***' 0.001 '**' 0.01 '*' 0.05 '.' 0.1 ' ' 1

ANOVA 2
Factors: Noise(<=0.0001)*Size*Propaganda(1/5,1/3,1/1):
Df Sum Sq Mean Sq F value               Pr(>F)
Noise                    1 0.0038 0.00378   1.489               0.2228
Size                     2 0.2294 0.11472  45.199 < 0.0000000000000002 ***
Propaganda               2 0.1112 0.05561  21.911       0.000000000516 ***
Noise:Size               2 0.0045 0.00227   0.896               0.4088
Noise:Propaganda         2 0.0062 0.00312   1.227               0.2936
Size:Propaganda          4 0.0203 0.00508   2.002               0.0923 .
Noise:Size:Propaganda    4 0.0015 0.00038   0.152               0.9623
Residuals              882 2.2387 0.00254
---
Signif. codes:  0 '***' 0.001 '**' 0.01 '*' 0.05 '.' 0.1 ' ' 1

ANOVA 3
Factors: Noise(<=0.0001)*Size(>=32x32)*Propaganda(1/5,1/3,1/1):
                       Df  Sum Sq Mean Sq F value               Pr(>F)
Noise                   1 0.00788 0.00788  18.563            0.0000193 ***
Size                    1 0.00682 0.00682  16.070            0.0000689 ***
Propaganda              2 0.12098 0.06049 142.552 < 0.0000000000000002 ***
Noise:Size              1 0.00008 0.00008   0.198               0.6561
Noise:Propaganda        2 0.00318 0.00159   3.744               0.0242 *
```

```
Size:Propaganda         2 0.00149 0.00074   1.750               0.1747
Noise:Size:Propaganda   2 0.00062 0.00031   0.735               0.4802
Residuals             588 0.24951 0.00042
---
Signif. codes:  0 '***' 0.001 '**' 0.01 '*' 0.05 '.' 0.1 ' ' 1


Averages of the compared groups
10x10:
    0.000001 0.00001 0.0001  0.001   0.01    0.1
1/1   0.0860  0.0978 0.0578 0.0216 0.9230 0.9022
1/3   0.0982  0.0924 0.0584 0.0126 0.1146 0.0394
1/5   0.1046  0.1052 0.0382 0.0144 0.0578 0.1606

32x32:
      0.000001    0.00001     0.0001      0.001       0.01        0.1
1/1 0.03835938 0.04074219 0.03046875 0.01876953 0.94769531 0.92603516
1/3 0.07048828 0.05957031 0.03685547 0.01375000 0.09410156 0.06259766
1/5 0.08279297 0.07183594 0.03351562 0.01503906 0.03740234 0.17296875

100x100:
    0.000001  0.00001   0.0001    0.001     0.01      0.1
1/1 0.052162 0.047994 0.033640 0.022672 0.949994 0.918334
1/3 0.075648 0.068164 0.035686 0.013492 0.057712 0.066692
1/5 0.086306 0.073970 0.029030 0.013694 0.035884 0.189448


Standard deviations of the compared groups
10x10:
      0.000001    0.00001     0.0001       0.001       0.01        0.1
1/1 0.06809357 0.10400726 0.04482574 0.023678114 0.06078567 0.03430297
1/3 0.08100617 0.07075569 0.04896313 0.007507819 0.15868349 0.02024442
1/5 0.08981182 0.07420985 0.04173238 0.011807988 0.03052533 0.04455723

32x32:
      0.000001    0.00001     0.0001      0.001        0.01         0.1
1/1 0.02060112 0.02536458 0.02052157 0.01645530 0.008467996 0.008671598
1/3 0.03042746 0.02191401 0.02558900 0.01464889 0.149118766 0.013177872
1/5 0.02856650 0.03017663 0.02040214 0.01200263 0.012138479 0.025101250

100x100:
      0.000001     0.00001      0.0001       0.001        0.01         0.1
1/1 0.01041206 0.014719769 0.006935740 0.018619510 0.005588706 0.003508858
1/3 0.01435031 0.010723097 0.012583533 0.008457260 0.011089279 0.004795229
1/5 0.01217529 0.009868301 0.009435436 0.007042739 0.007500676 0.014852035
```

27
