# Supplementary material for: Institutions and Cultural Diversity: Effects of Democratic and Propaganda Processes on Local Convergence and Global Diversity
Source: PLoS One. 2016 Apr 8;11(4):e0153334. doi: 10.1371/journal.pone.0153334 (PMC4825973; doi:10.1371/journal.pone.0153334)
Supplement: S5 File — (PDF) [file pone.0153334.s005.pdf]

S5 File. Stable states of equilibrium.

Fig. A to Fig. F display convergence in our experiment A models for all 6 different levels of noise (Fig. A starting with 0.000001 to Fig. F at 0.1). Each line displays (as an average over 50 repetitions) the behavior of the systems as the agents attempt on average 100,000 interactions with their neighbours. The end point of each line (at 100,000 interactions) corresponds to one single point in Fig. 3 in the main paper.

To summarize, we can see that some configurations of alpha = 0.5 do not reach an equilibrium, so they may further converge towards a monoculture. Less extreme, but similarly the lines at alpha = 0.95 do not seem to have reached complete stability yet, and some decrease is to be expected. For all other values and noise levels, the level of stability is satisfactory.

Fig. G shows single runs from the same configurations as above. Each run was randomly selected out of the 50 repetitions that we performed. Comparing Fig. G to L to Fig. A to F, we can say that our averaged lines are good representations of individual lines' behaviors.

Cultural regions over time at n=0.000001

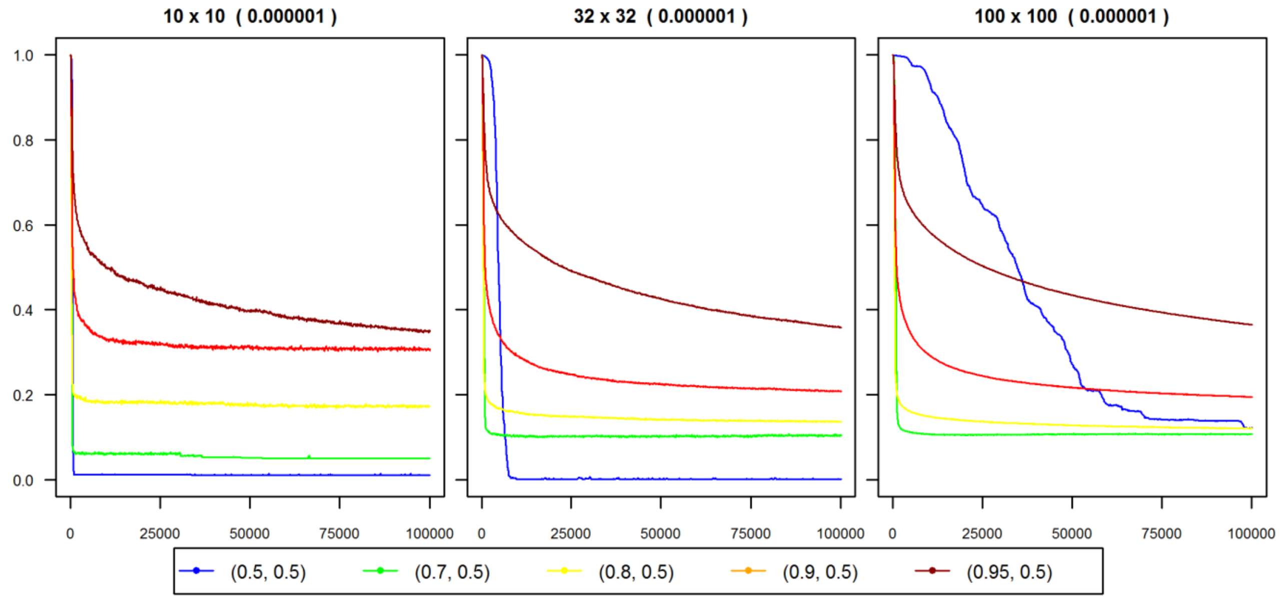

Fig. A. Average number of cultural regions over time with n=0.000001. The averages are calculated out of 50 repetitions. Axis-x represent the iteration number, and Axis-Y the cultural diversity (number of cultures divided by population size). From left to right, the graph represents 10x10, 32x32 and 100x100 populations. The legend shows the color use for the different values of alpha (institutional influence).

Cultural regions over time at n=0.00001

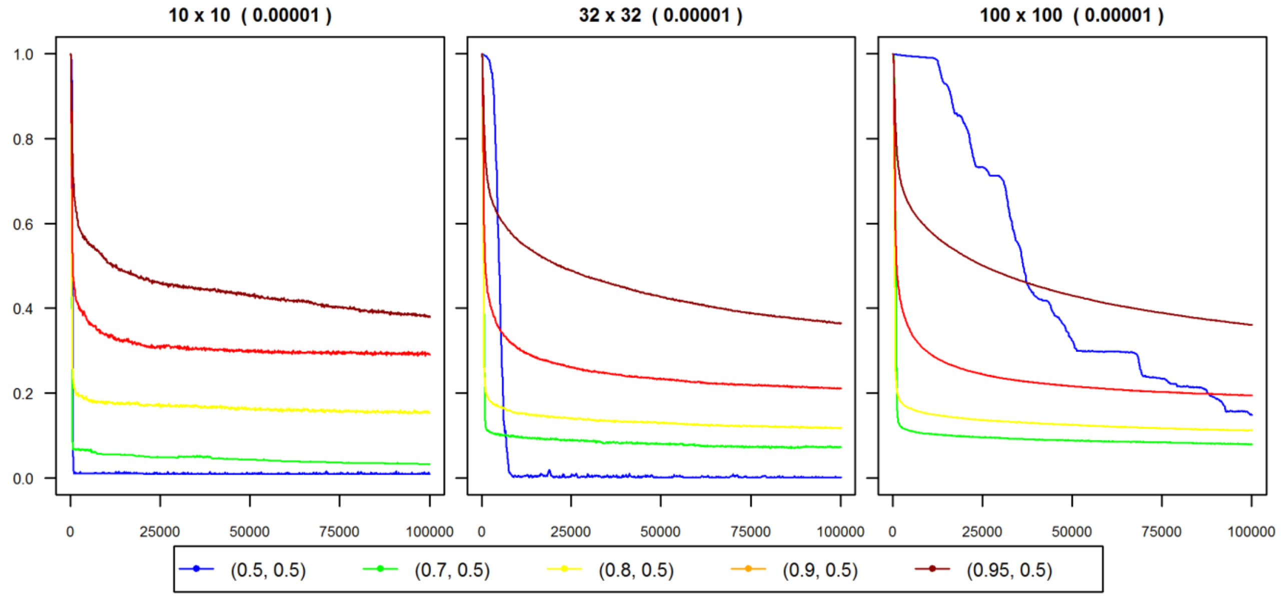

Fig. B. Average number of cultural regions over time with n=0.00001. The averages are calculated out of 50 repetitions. Axis-x represent the iteration number, and Axis-Y the cultural diversity (number of cultures divided by population size). From left to right, the graph represents 10x10, 32x32 and 100x100 populations. The legend shows the color use for the different values of alpha (institutional influence).

Cultural regions over time at n=0.0001

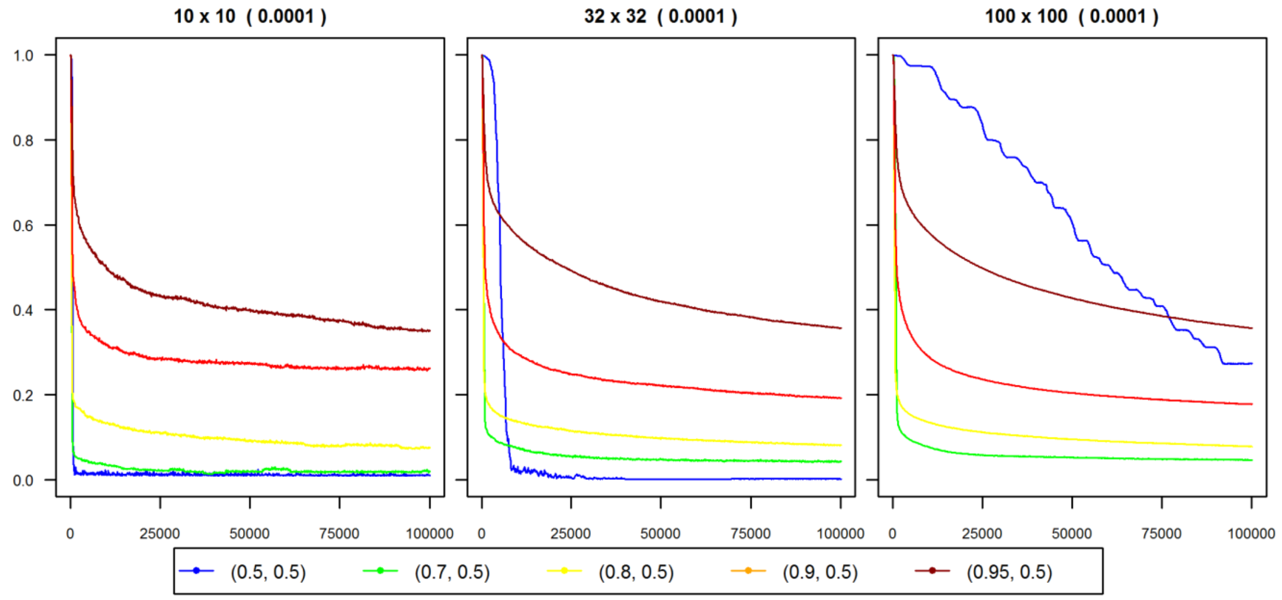

Fig. C. Average number of cultural regions over time with n=0.0001. The averages are calculated out of 50 repetitions. Axis-x represent the iteration number, and Axis-Y the cultural diversity (number of cultures divided by population size). From left to right, the graph represents 10x10, 32x32 and 100x100 populations. The legend shows the color use for the different values of alpha (institutional influence).

Cultural regions over time at n=0.001

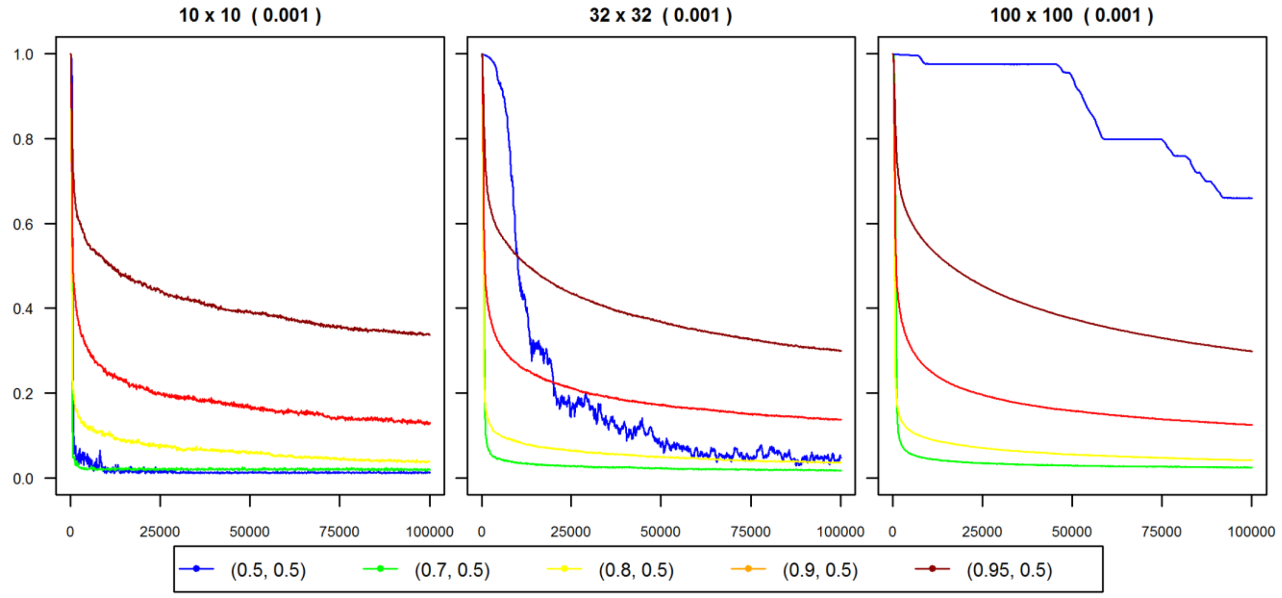

Fig. D. Average number of cultural regions over time with n=0.001. The averages are calculated out of 50 repetitions. Axis-x represent the iteration number, and Axis-Y the cultural diversity (number of cultures divided by population size). From left to right, the graph represents 10x10, 32x32 and 100x100 populations. The legend shows the color use for the different values of alpha (institutional influence).

Cultural regions over time at n=0.01

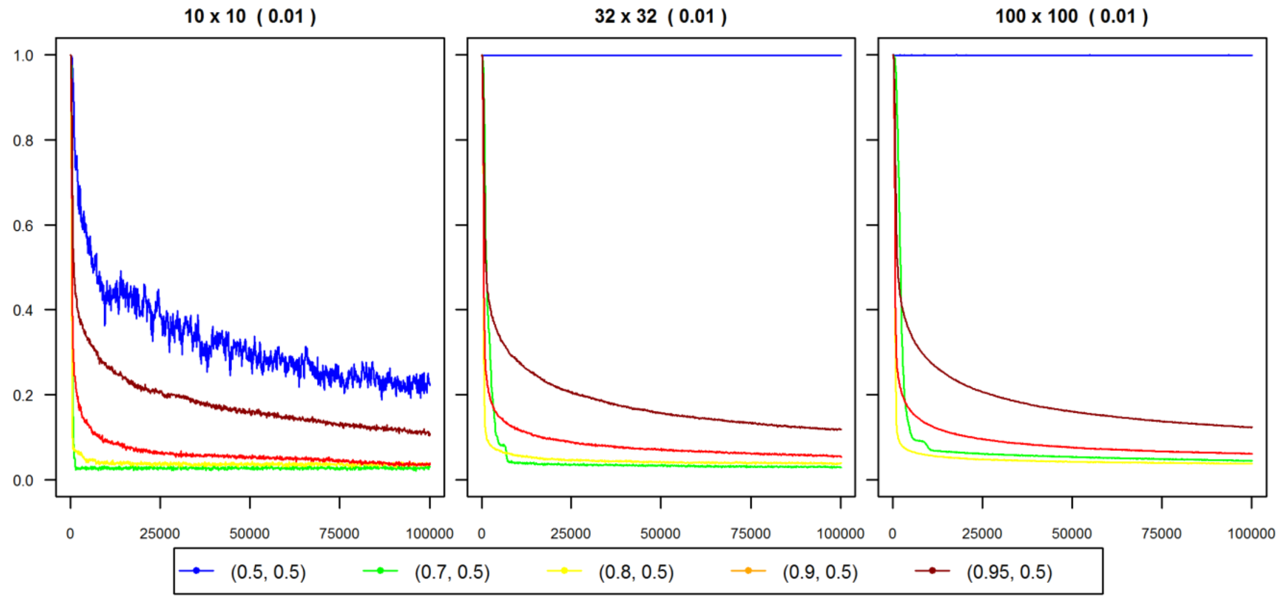

Fig. E. Average number of cultural regions over time with n=0.01. The averages are calculated out of 50 repetitions. Axis-x represent the iteration number, and Axis-Y the cultural diversity (number of cultures divided by population size). From left to right, the graph represents 10x10, 32x32 and 100x100 populations. The legend shows the color use for the different values of alpha (institutional influence).

Cultural regions over time at n=0.1

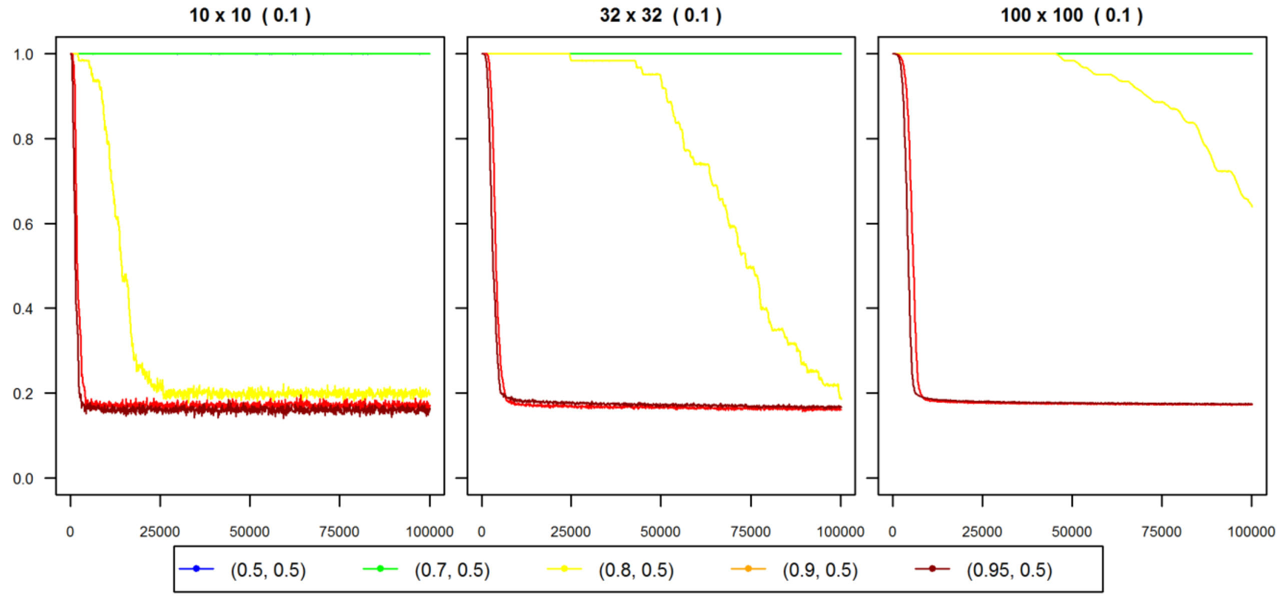

Fig. F. Average number of cultural regions over time with n=0.1. The averages are calculated out of 50 repetitions. Axis-x represent the iteration number, and Axis-Y the cultural diversity (number of cultures divided by population size). From left to right, the graph represents 10x10, 32x32 and 100x100 populations. The legend shows the color use for the different values of alpha (institutional influence).

Cultural regions over time for one run at n=0.000001

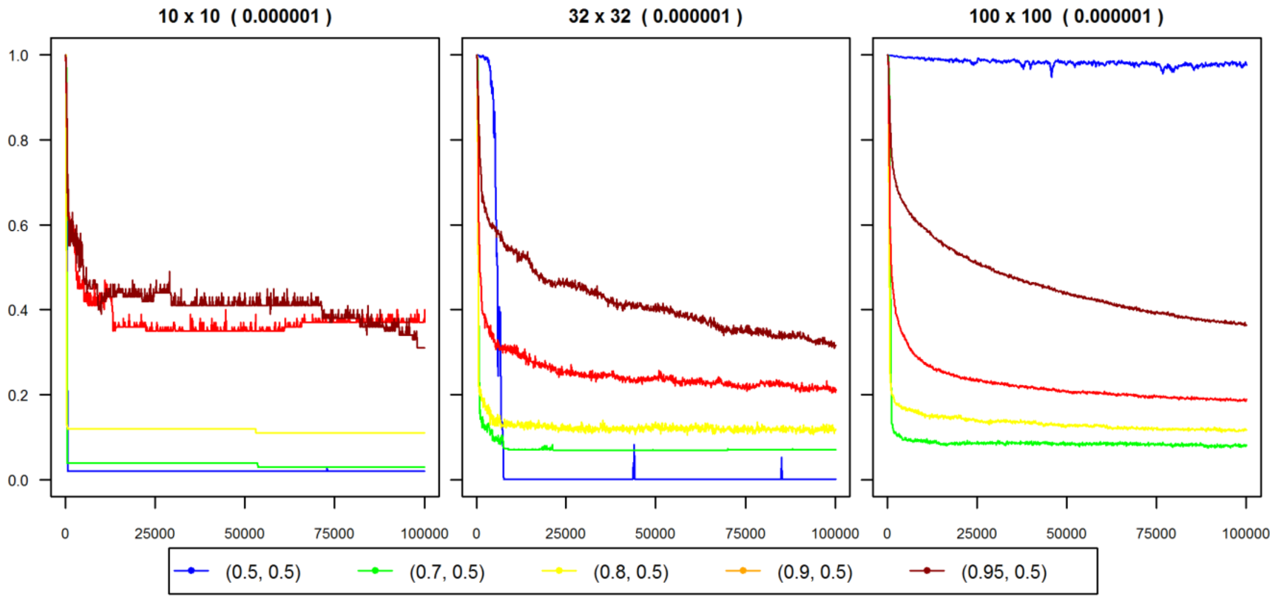

Fig. G. Number of cultural regions over time for single runs with n=0.000001. Each line represent a single run of the simulation. Axis-x represent the iteration number, and Axis-Y the cultural diversity (number of cultures divided by population size). From left to right, the graph represents 10x10, 32x32 and 100x100 populations. The legend shows the color use for the different values of alpha (institutional influence).

Cultural regions over time for one run at n=0.00001

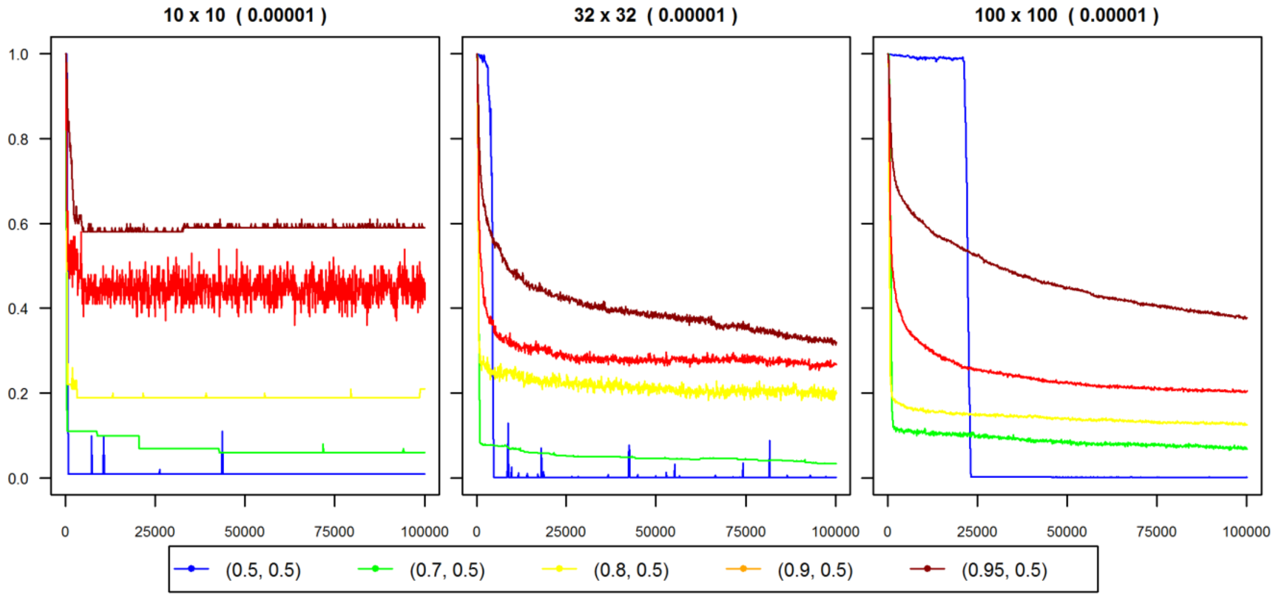

Fig. H. Number of cultural regions over time for single runs with n=0.00001. Each line represent a single run of the simulation. Axis-x represent the iteration number, and Axis-Y the cultural diversity (number of cultures divided by population size). From left to right, the graph represents 10x10, 32x32 and 100x100 populations. The legend shows the color use for the different values of alpha (institutional influence).

Cultural regions over time for one run at n=0.0001

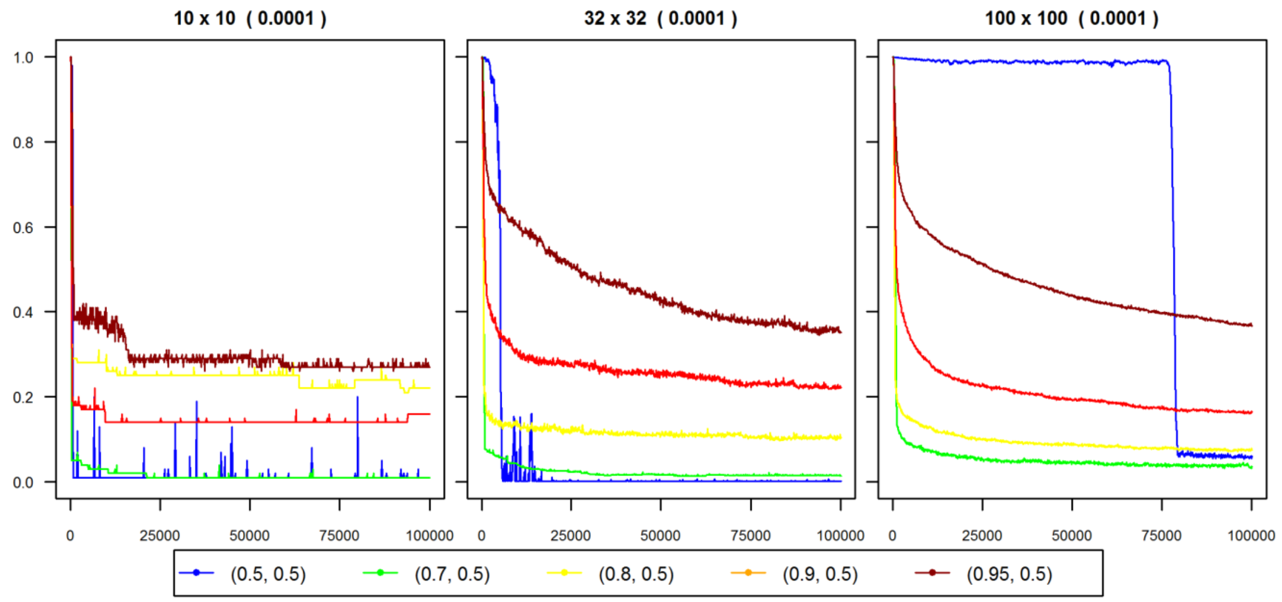

Fig. I. Number of cultural regions over time for single runs with n=0.0001. Each line represent a single run of the simulation. Axis-x represent the iteration number, and Axis-Y the cultural diversity (number of cultures divided by population size). From left to right, the graph represents 10x10, 32x32 and 100x100 populations. The legend shows the color use for the different values of alpha (institutional influence).

Cultural regions over time for one run at n=0.001

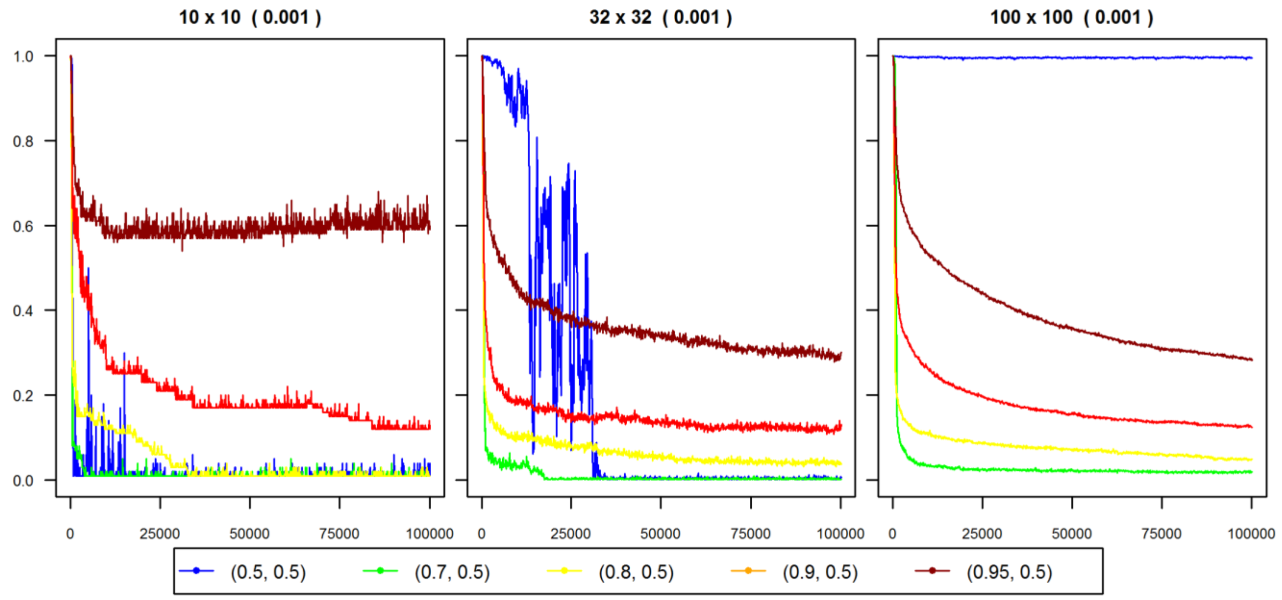

Fig. J. Number of cultural regions over time for single runs with n=0.001. Each line represent a single run of the simulation. Axis-x represent the iteration number, and Axis-Y the cultural diversity (number of cultures divided by population size). From left to right, the graph represents 10x10, 32x32 and 100x100 populations. The legend shows the color use for the different values of alpha (institutional influence).

Cultural regions over time for one run at n=0.01

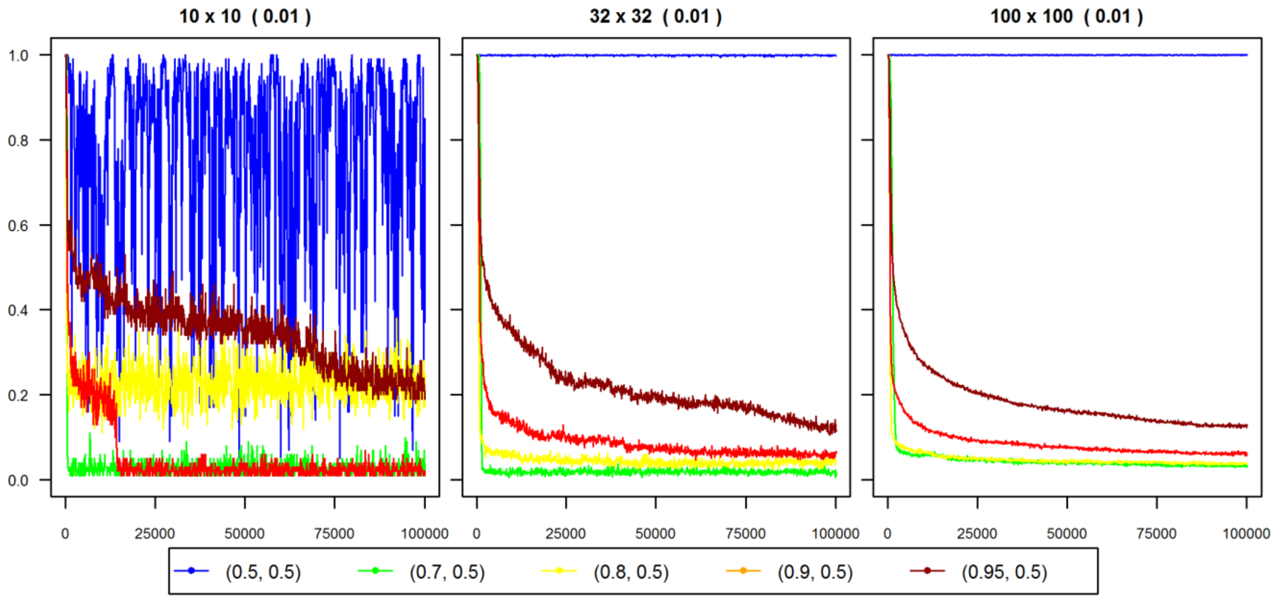

Fig. K. Number of cultural regions over time for single runs with n=0.01. Each line represent a single run of the simulation. Axis-x represent the iteration number, and Axis-Y the cultural diversity (number of cultures divided by population size). From left to right, the graph represents 10x10, 32x32 and 100x100 populations. The legend shows the color use for the different values of alpha (institutional influence).

Cultural regions over time for one run at n=0.1

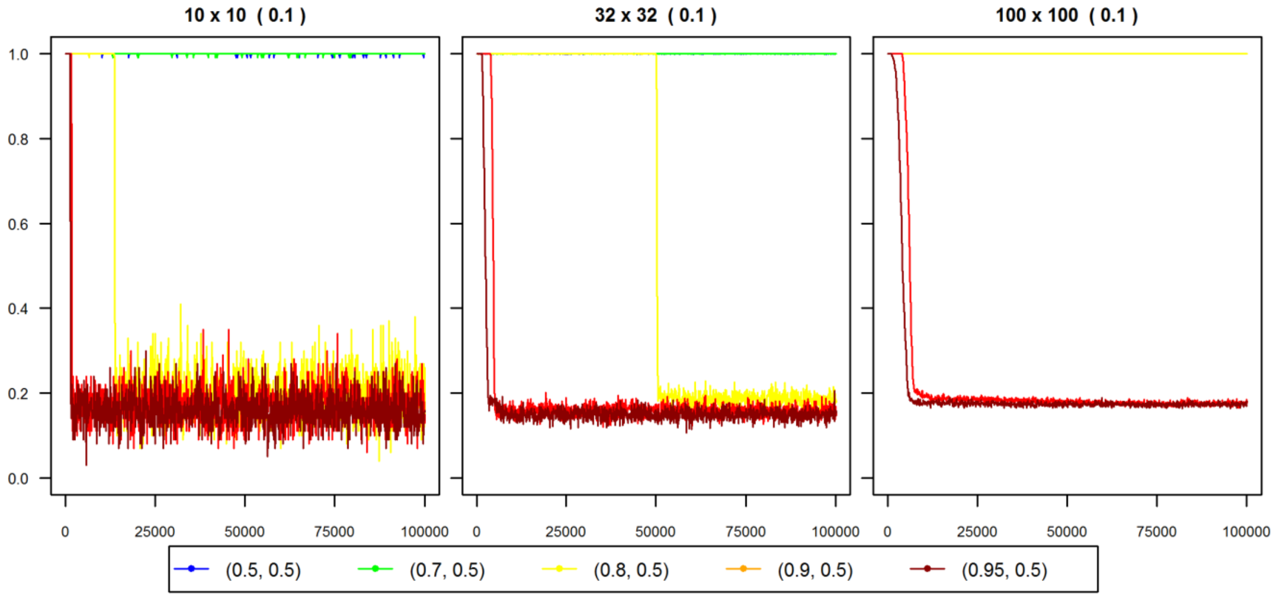

Fig. K. Number of cultural regions over time for single runs with n=0. 1. Each line represent a single run of the simulation. Axis-x represent the iteration number, and Axis-Y the cultural diversity (number of cultures divided by population size). From left to right, the graph represents 10x10, 32x32 and 100x100 populations. The legend shows the color use for the different values of alpha (institutional influence).
