# Supplementary material for: Institutions and Cultural Diversity: Effects of Democratic and Propaganda Processes on Local Convergence and Global Diversity
Source: PLoS One. 2016 Apr 8;11(4):e0153334. doi: 10.1371/journal.pone.0153334 (PMC4825973; doi:10.1371/journal.pone.0153334)
Supplement: S6 File — (PDF) [file pone.0153334.s006.pdf]

S6 File. Replication of Axelrod/Flache's results.

We will now present results from a replication of both Axelrod's and Flache's models with Flache's implementation comparing them to our own code implementation.

We decided to re-implement Flache's model because:

(1) we have the intention to integrate our model of institutional influence with Flache's model of multilateral social influence in the future
(2) because when we attempted a replication of their model with their code, we had computational failures, especially for the biggest population size, which requires a lot of computational power

We optimized the code in several ways:

(1) we do not implement a graphical user interface
(2) we introduce thread management
(3) we use native matrices
(4) we avoid the use of classes, methods and unnecessary initializations (recycling structures)
(5) we use buffers to manage the input and output of results

The replication of their model with our code was qualitatively successful (see continuous line in Fig. , where the lines in the plot are almost parallel and converge with higher values of noise). However, statistically, we did find a significant difference between the two implementations. Table 1 displays the ANOVA with three factors: implementation (i.e. two code variations), population size, and noise. The test is limited to only the two population sizes 10x10 and 32x32, because as mentioned before, due to computational difficulties we could not finish a run with their code for the population 100x100. Finally, our replications yields better results for our implementation of Flache et al's model, i.e. we manage to produce more diversity for their hypothesis (on their response variable, size of the biggest culture) with our code than when implementing theirs. This means that testing our data against theirs for comparison, we err on the side of conservatism.

Table 1. Anova comparing the two implementations (Implem), Flache's code vs our code, population size (N) and noise. The implementations are two replications of the multilateral social influence model proposed by Flache et al, one is Flache's code implementation, and the other is ours.

```
Anova Table (Type I tests)
Response: Size of the biggest culture
Factors: Size*Noise*Implem
                    Df  Sum Sq Mean Sq  F value               Pr(>F)
Size                 1 2451919 2451919 8856.959 < 0.0000000000000002 ***
Noise                5  626111  125222  452.335 < 0.0000000000000002 ***
Implem               1   22283   22283   80.491 < 0.0000000000000002 ***
Size:Noise           5  346196   69239  250.109 < 0.0000000000000002 ***
Size:Implem          1   12526   12526   45.247      0.0000000000271 ***
Noise:Implem         5   14355    2871   10.371      0.0000000009474 ***
Size:Noise:Implem    5    7693    1539    5.558      0.0000455284940 ***
Residuals         1176  325558     277
---
Signif. codes:  0 '***' 0.001 '**' 0.01 '*' 0.05 '.' 0.1 ' ' 1


Averages of the compared groups
Flache's implementation:
     0.000001 0.00001 0.0001 0.001  0.01    0.1
100     16.58   16.24  12.18  6.18  2.74  10.36
1024   130.26  131.36 105.12 65.16 34.14 101.90

Our implementation:
     0.000001 0.00001 0.0001 0.001  0.01   0.1
100     22.82    19.3  14.76  7.54  2.40  10.4
1024   157.84   158.2 131.62 69.88 36.38 104.5


Standard deviations of the compared groups
Flache's implementation:
     0.000001  0.00001    0.0001     0.001     0.01       0.1
100  10.45123 10.09457  8.416917  7.702875 2.655837  2.553589
1024 28.34252 27.02437 26.042070 18.505774 8.111493 10.842565

Our implementation:
     0.000001   0.00001    0.0001     0.001     0.01       0.1
100  10.73938  7.442981 10.62642  8.048856 1.428571  3.103652
1024 27.28486 29.686113 30.83524 20.800942 8.243464 10.689018
```

Graph of biggest cultural regions comparing Axelrod and Flache in Flaches code vs our code

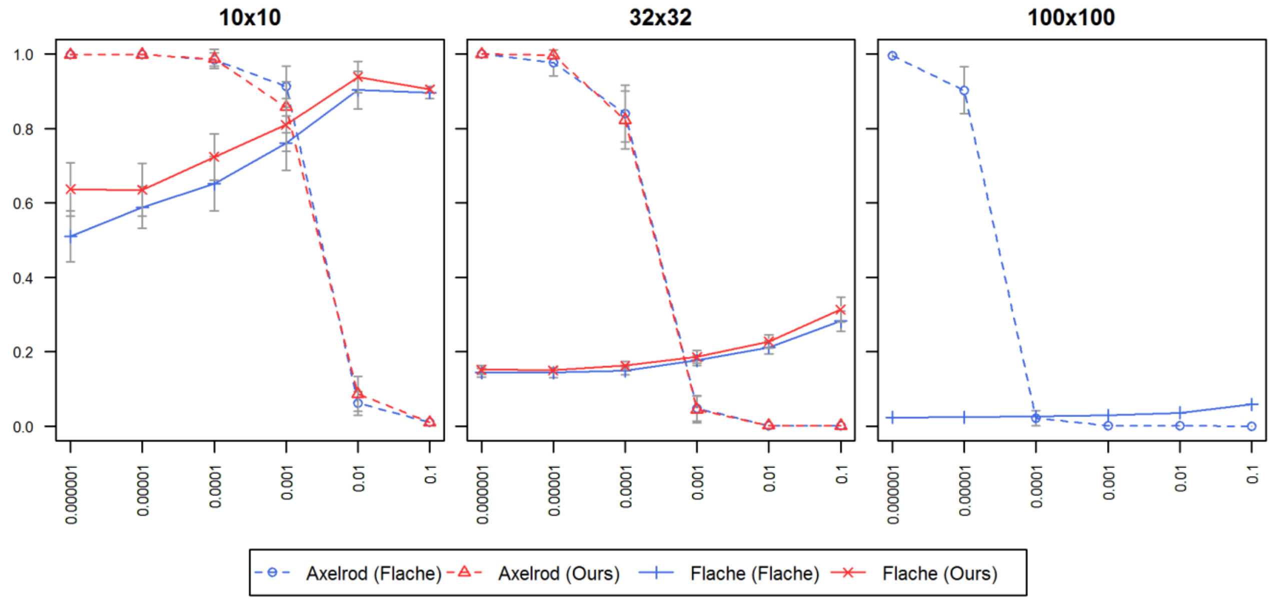

Fig. A Biggest cultural region on different implementation of the same model. The X axis shows the different levels of noise, and the Y axis the normalized size of the biggest cultural region (size of the biggest cultural region/population size). The results of the two different models (Axelrod and Flache), as implement by Flache (blue) vs ours (red) are represented in the graph for populations 10x10 and 32x32, while only Flache's code (for Axelrod and Flache) is displayed in 100x100. Each point on the line is an average of the final cultural diversity of 50 repetitions with 100000 iterations on average per agent. Confidence intervals at 0.95 are displayed only when bigger than the identifier symbols.

For this comparison, we are displaying size of the biggest culture, just as Flache did in his original paper. Flache opted for an indirect measurement that allows best for observation of the stability of the system against noise, i.e. whether there is a tendency towards globalization or anomie. Globalization is reached when the biggest culture absorbs all the agents in the population; similarly, anomie is reached when the biggest culture consists of one agent. Since we were more interested in the effects of institutions on actual cultural diversity rather than the stability of the system, we decided to keep the number of cultural regions to denominate cultural diversity as the main response variable across the paper. However, we are presenting the graphs using their response variable in this Appendix section.

Before that, we also show our implementation of Axelrod's model, and include the two noise sources as described by Flache. Fig. A displays our results here in dotted lines. We found no

significant differences between Axelrod's model with Flache's code and our replication, as seen in Table 2.

Table 2. Anova comparing the two implementations (Implem), Axelrode with Flache's code vs our code, population size (N) and noise. The implementations are two replications of the dyadic social influence model proposed by Axelrod, one is Flache's code implementation, and the other is ours.

```
Anova Table (Type I tests)
Response: Size of the biggest culture
                      Df   Sum Sq  Mean Sq   F value               Pr(>F)
Size                   1 63001127 63001127 13974.324 <0.0000000000000002 ***
Noise                  5 76508238 15301648  3394.069 <0.0000000000000002 ***
Implem                 1       30       30     0.007                0.935
Size:Noise             5 56680215 11336043  2514.456 <0.0000000000000002 ***
Size:Implem            1      177      177     0.039                0.843
Noise:Implem           5     2113      423     0.094                0.993
Size:Noise:Implem      5     1898      380     0.084                0.995
Residuals           1176  5301818     4508
---
Signif. codes:  0 '***' 0.001 '**' 0.01 '*' 0.05 '.' 0.1 ' ' 1

Averages of the compared groups
Flache's implementation:
     0.000001 0.00001 0.0001  0.001    0.01     0.1
100      1.14    1.06   1.36   7.68   83.18   99.98
1024     1.00    2.88  80.98 817.08 1022.64 1024.00

Our implementation
     0.000001 0.00001 0.0001  0.001    0.01     0.1
100      1.12    1.10   1.84   5.24   87.82   99.98
1024     1.00    8.18  70.50 815.38 1023.08 1023.92


Standard deviations of the compared groups
Flache's implementation:
      0.000001   0.00001     0.0001     0.001      0.01       0.1
100  0.4952839 0.2398979   2.405436  10.02332 20.430759 0.1414214
1024 0.0000000 9.5096943 117.053187 197.68619  1.224911 0.0000000

Our implementation:
      0.000001    0.00001     0.0001      0.001       0.01       0.1
100  0.3282607  0.5802885   3.253632   7.487568 15.8393594 0.1414214
1024 0.0000000 26.3234899 116.409946 200.599498  0.8533248 0.2740475
```

Results graphs with response variable "size of biggest culture"

We will now show all our results graphs as presented in the main paper, adopting Flache's chosen variable of "size of the biggest cultural region", to clarify that there were no meaningful differences even if we had used that variable instead of the one we chose. Fig. B to Fig. G display the results of Experiment A to F.

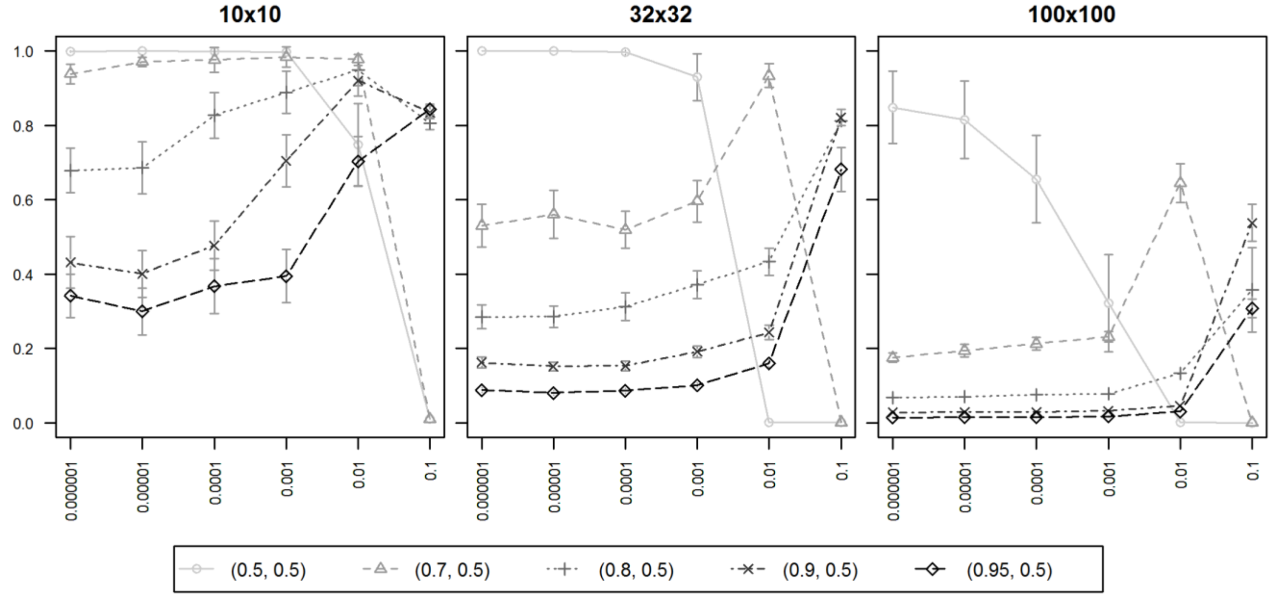

Fig. B. Size of the biggest culture for varying levels of institutional influence. X-axis displays levels of noise; Y axis displays biggest culture. Each line symbol denotes one alpha of institutional influence. 95% confidence intervals are displayed only when exceeding the size of the line symbol. Data points are averages of 50 replications per territory with 100,000 iterations per agent.

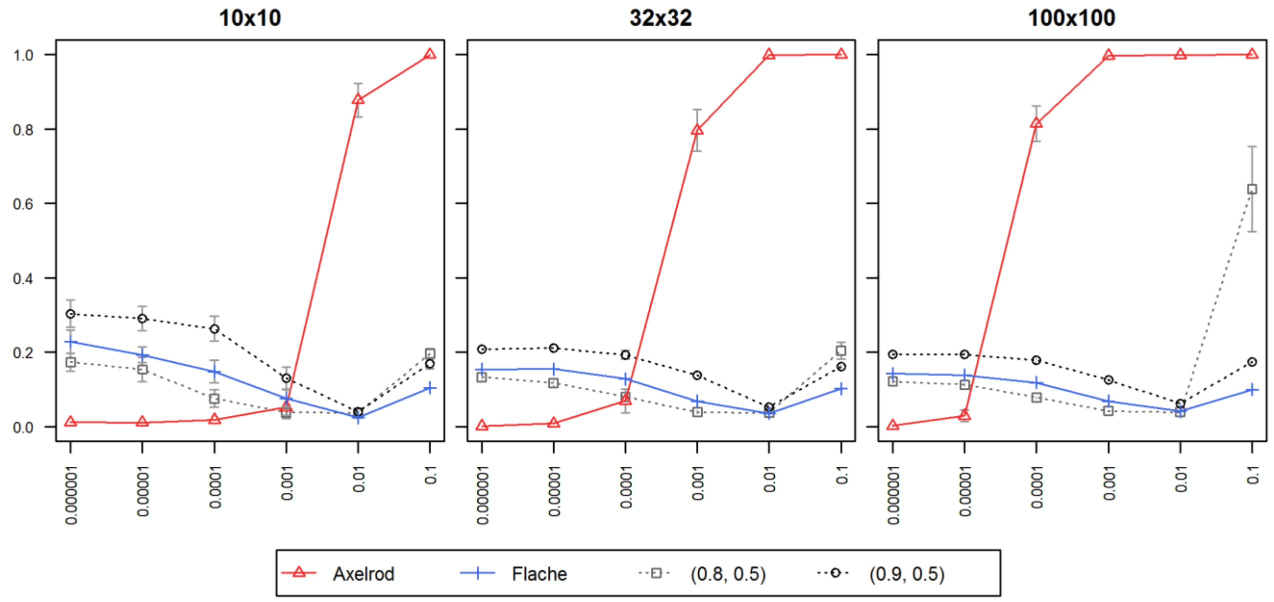

Fig. C. Size of the biggest culture for comparison with Axelrod and Flache. X-axis displays levels of noise; Y axis displays biggest culture. Each line symbol denotes the models we chose as comparisons. 95% confidence intervals are displayed only when exceeding the size of the line symbol. Data points are averages of 50 replications per territory with 100,000 iterations per agent.

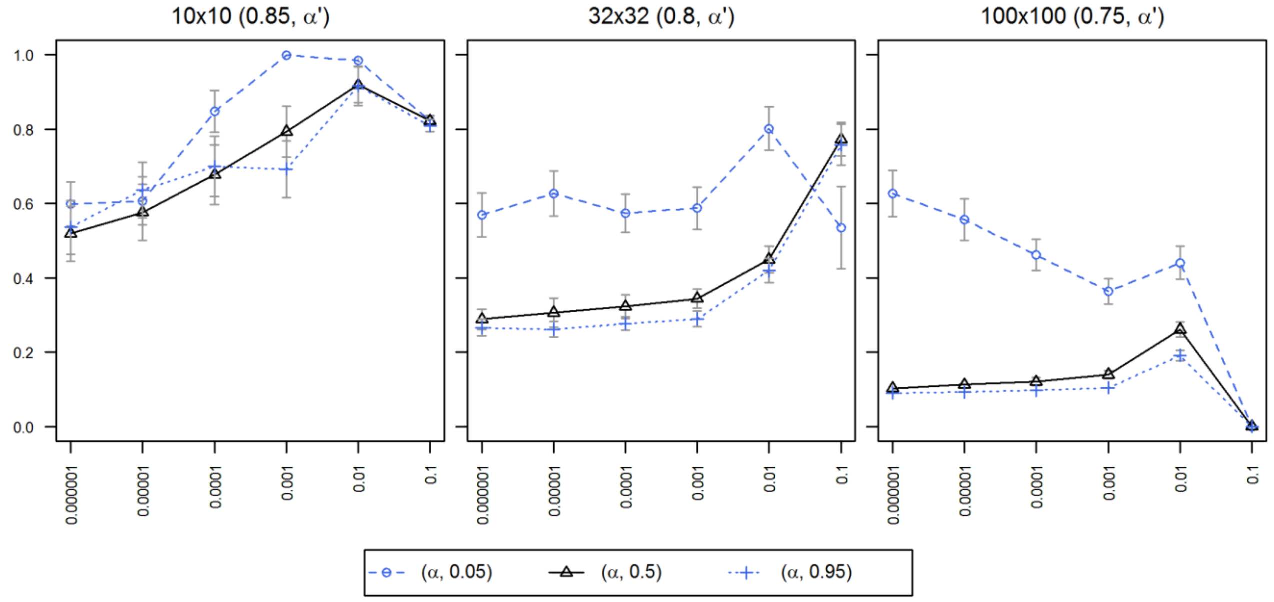

Fig. D. Size of the biggest culture for varying levels of agent loyalty. X-axis displays levels of noise; Y axis displays biggest culture. Each line symbol denotes one alpha prime of agent loyalty. 95% confidence intervals are displayed only when exceeding the size of the line symbol. Data points are averages of 50 replications per territory with 100,000 iterations per agent.

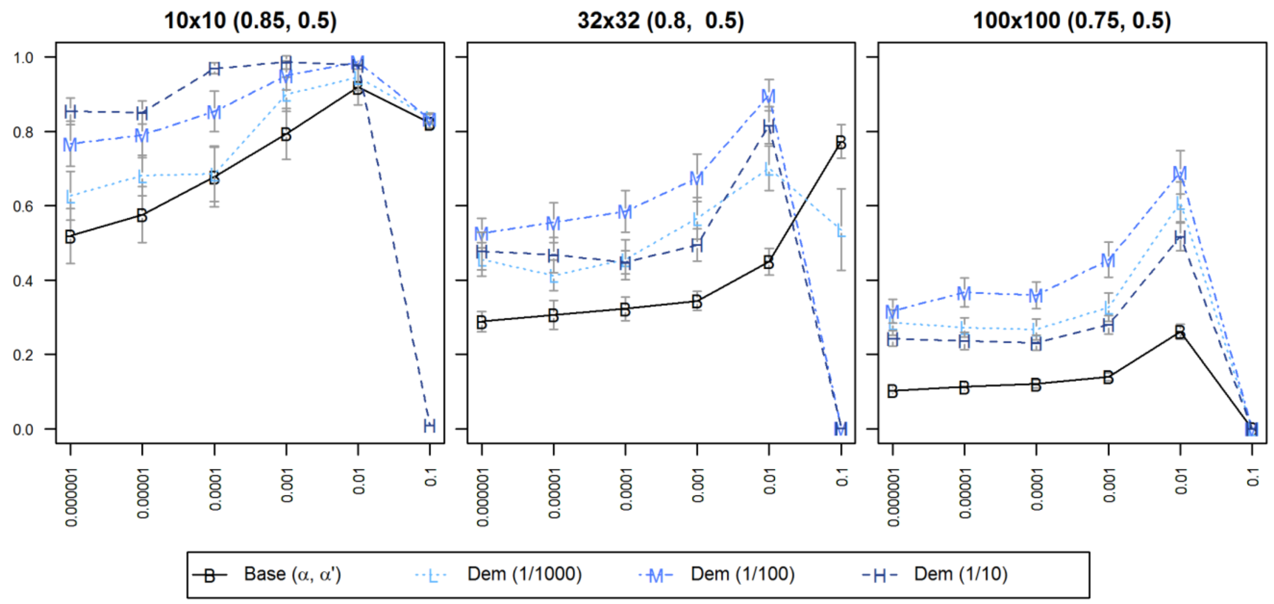

Fig. E. Size of the biggest culture for varying frequencies of democracy. X-axis displays levels of noise; Y axis displays biggest culture. Each line symbol denotes frequency of democracy. 95% confidence intervals are displayed only when exceeding the size of the line symbol. Data points are averages of 50 replications per territory with 100,000 iterations per agent.

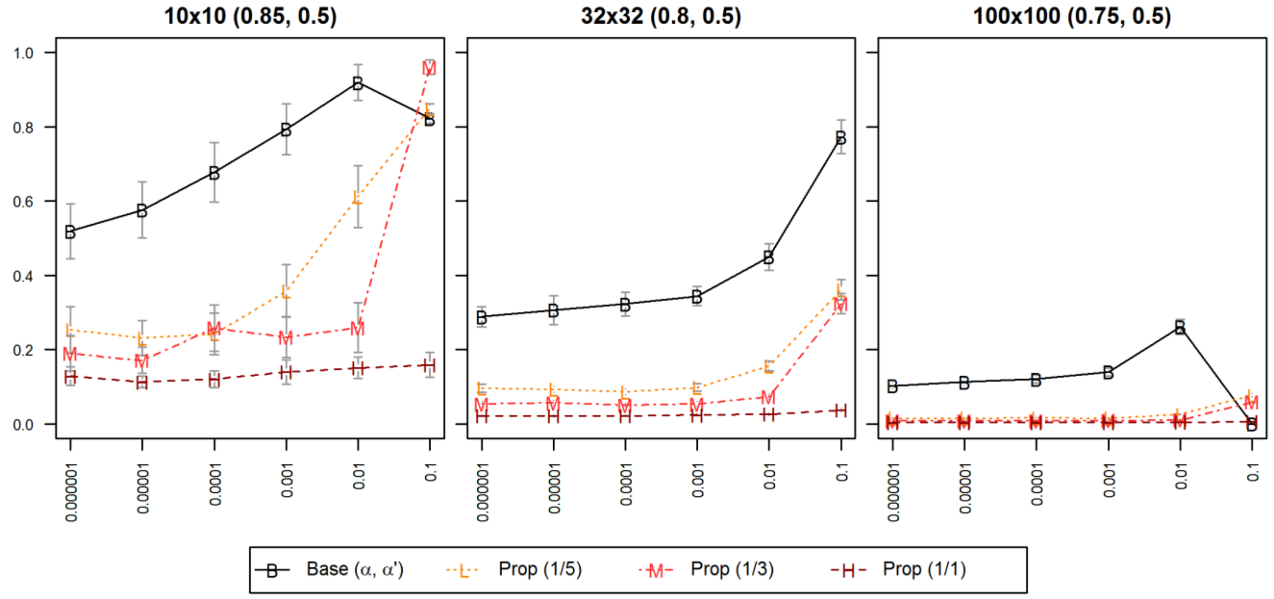

Fig. F. Size of the biggest culture for varying frequency of propaganda. X-axis displays levels of noise; Y axis displays biggest culture. Each line symbol denotes one frequency of propaganda. 95% confidence intervals are displayed only when exceeding the size of the line symbol. Data points are averages of 50 replications per territory with 100,000 iterations per agent.

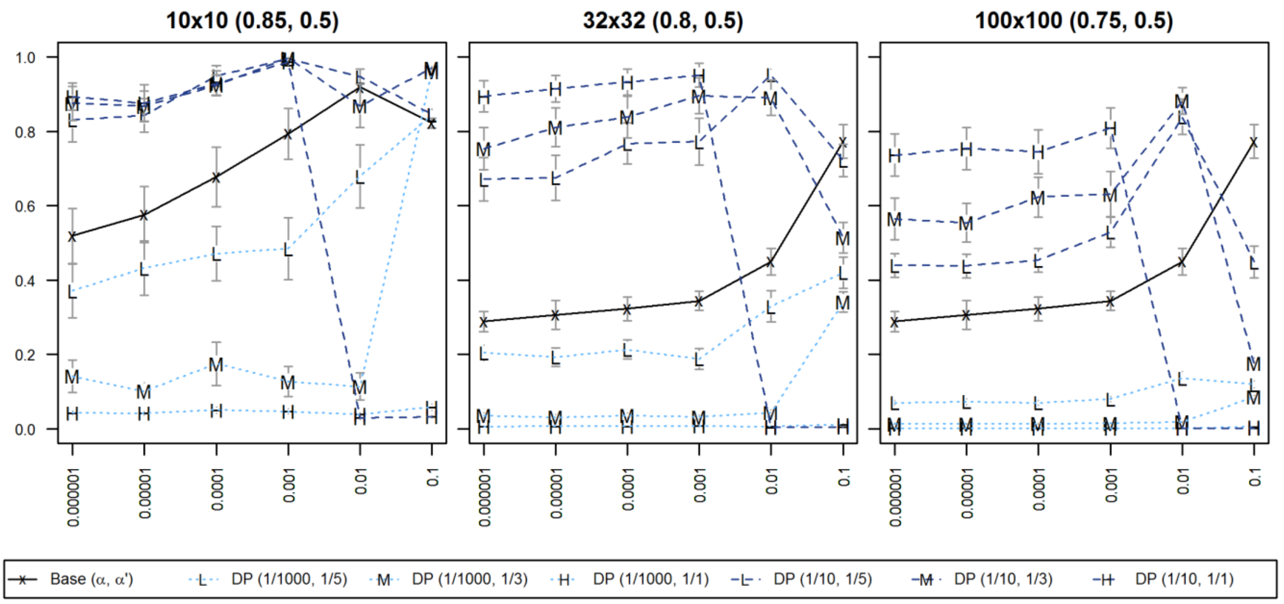

Fig. G. Size of the biggest culture for combined democracy and propaganda frequencies. X-axis displays levels of noise; Y axis displays biggest culture. Each line symbol denotes one combination of democracy and propaganda frequencies. 95% confidence intervals are displayed only when exceeding the size of the line symbol. Data points are averages of 50 replications per territory with 100,000 iterations per agent.
