# Supplementary material for: Institutions and Cultural Diversity: Effects of Democratic and Propaganda Processes on Local Convergence and Global Diversity
Source: PLoS One. 2016 Apr 8;11(4):e0153334. doi: 10.1371/journal.pone.0153334 (PMC4825973; doi:10.1371/journal.pone.0153334)
Supplement: S7 File — (PDF) [file pone.0153334.s007.pdf]

S7 File. Number of institutions.

Fig. A to Fig. D give an overview over the number of institutions that resulted in our systems in experiments A (institutional influence), C (agents loyalty), D (democracy) and E (propaganda). The results of experiment F (democracy + propaganda) are discussed in the main paper.

Fig. A (from experiment A) confirms the observations from Table 4 of the main paper, i.e. the number of institution is proportional to the population size (the values in the graph are normalized by population), and they are not strongly affected by institutional influence. Similarly, Fig. B (from experiment C) shows that the number of institutions doesn't seem to be affected by agent loyalty, except for models 100x100/0.75/0.05, only when noise is low (<=0.001).

Conversely, Fig. C and Fig. D show that increasing democracy and propaganda leads to an increase in the number of institutions in either case. These graphs are consistent with Fig. 9 and its associated discussion in the main paper, although the effects of propaganda seems to be amplified by democracy when the two institutional processes are combined.

Results graphs with response variable "number of institutions"

Fig. A to **Fig. B** displays the normalized number of institutions for Experiments A, B, D and F. This is to complement the Fig 9. (main paper). **Fig. A** mainly confirm observations made in Experiment A regarding to Table 4, however the scale is to small to appreciate the effects. **Fig. B** shows no relevant information for institution loyalty. **Fig. C** shows how democracy promotes the preservation of institutions. **Fig. D** shows a similar, but weaker effect, for propaganda. This results are the same as observed in Fig 9, when both democracy and propaganda are present.

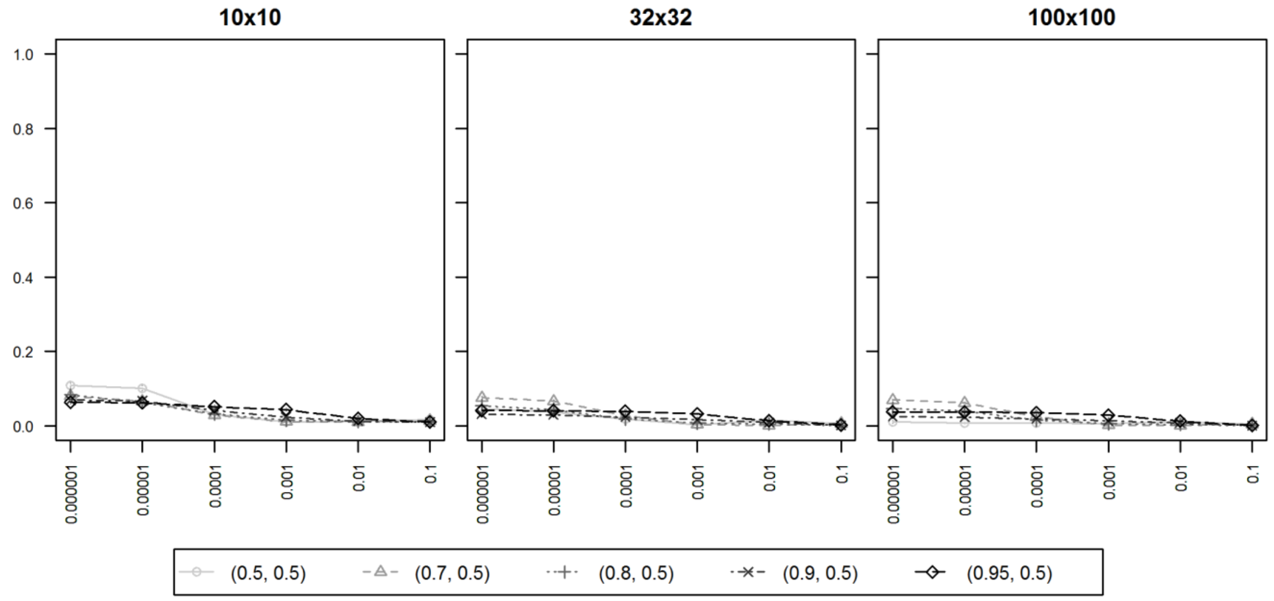

Fig. A. Number institutions for varying levels of institutional influence. X-axis displays levels of noise; Y axis displays normalized number of institutions. Each line symbol denotes one alpha of institutional influence. 95% confidence intervals are displayed only when exceeding the size of the line symbol. Data points are averages of 50 replications per territory with 100,000 iterations per agent.

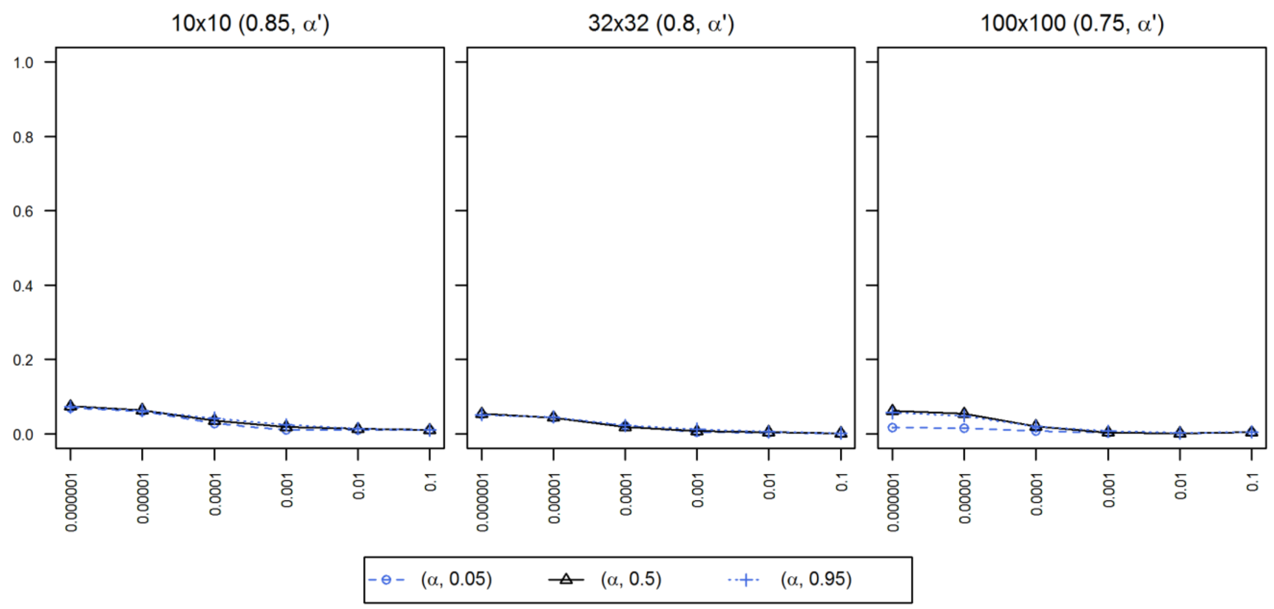

Fig. B. Number institutions for varying levels of agent loyalty. X-axis displays levels of noise; Y axis displays normalized number of institutions. Each line symbol denotes one alpha prime of agent loyalty. 95% confidence intervals are displayed only when exceeding the size of the line symbol. Data points are averages of 50 replications per territory with 100,000 iterations per agent.

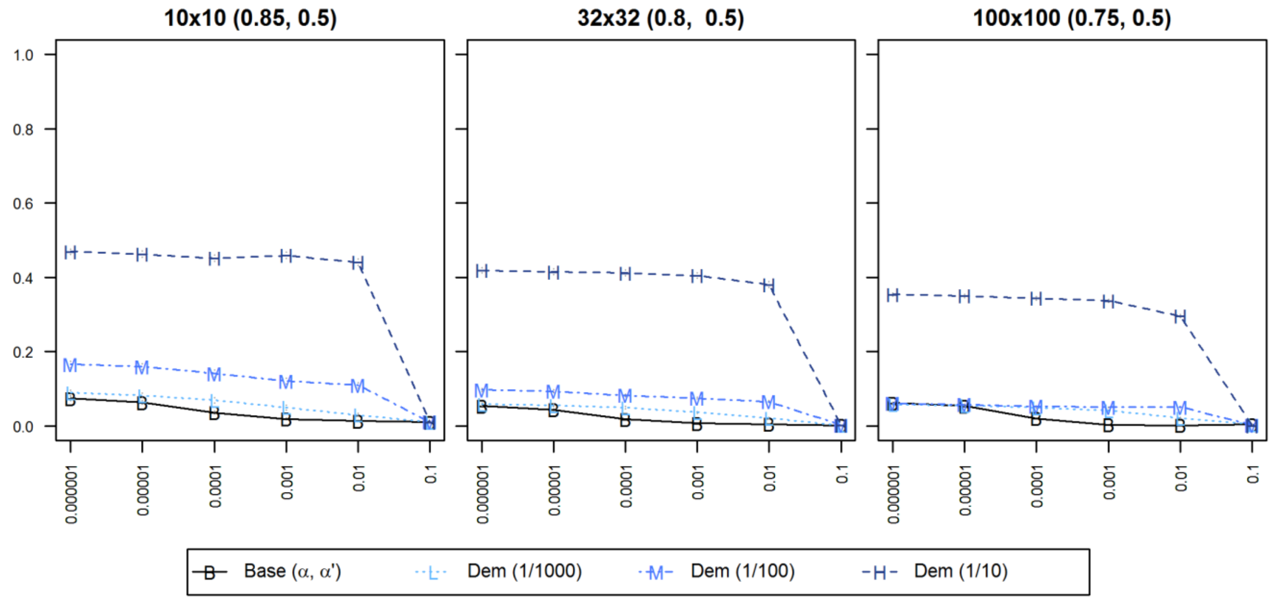

Fig. C. Number institutions for varying frequency of democracy. X-axis displays levels of noise; Y axis displays normalized number of institutions. Each line symbol denotes one frequency of democracy. 95% confidence intervals are displayed only when exceeding the size of the line symbol. Data points are averages of 50 replications per territory with 100,000 iterations per agent.

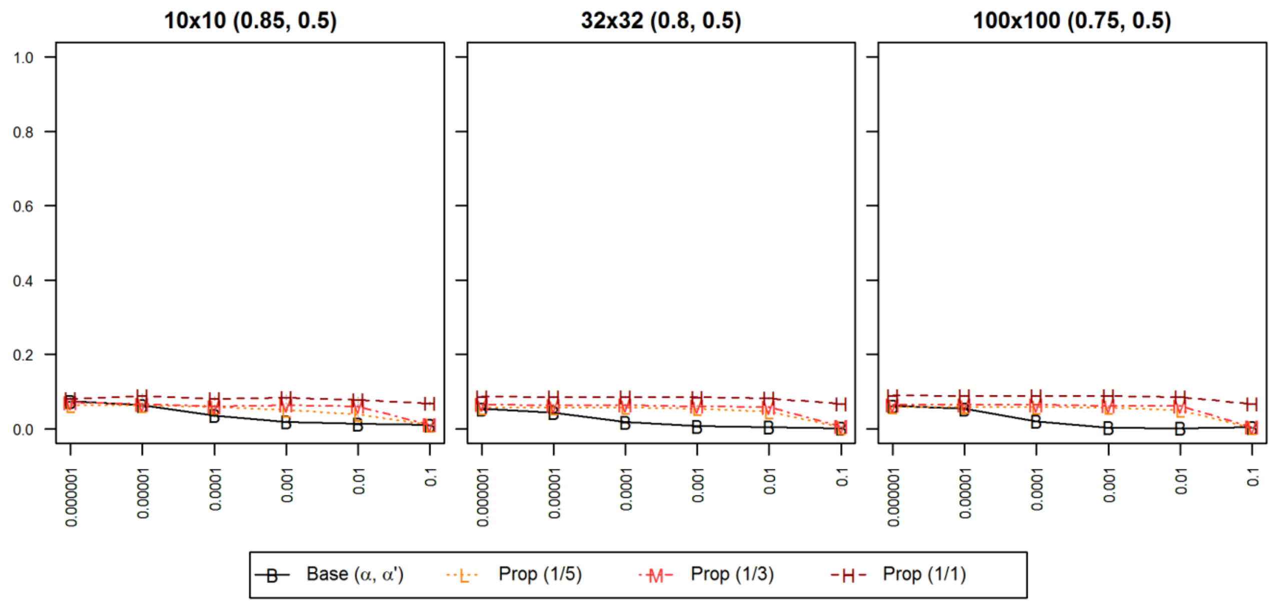

Fig. D. Number institutions for frequencies of propaganda. X-axis displays levels of noise; Y axis displays normalized number of institutions. Each line symbol denotes one frequency of institutions. 95% confidence intervals are displayed only when exceeding the size of the line symbol. Data points are averages of 50 replications per territory with 100,000 iterations per agent.
