# Supplementary material for: Institutions and Cultural Diversity: Effects of Democratic and Propaganda Processes on Local Convergence and Global Diversity
Source: PLoS One. 2016 Apr 8;11(4):e0153334. doi: 10.1371/journal.pone.0153334 (PMC4825973; doi:10.1371/journal.pone.0153334)
Supplement: S8 File — (PDF) [file pone.0153334.s008.pdf]

S8 File. Complete results Experiment F (inclusion of democracy 1/100).

Fig. A displays the complete results for Experiment F, i.e. it includes the medium values of democracy (1/100). The results are represented with the dashed-dotted lines. We can observe that the lines are located between the high (1/10) and low (1/1000) values of democracy, with a few exceptions for high values of noise (>= 0.01). The only big effect we found for medium democracy (1/100) was at the highest frequency of propaganda (1/1).

In general the results confirmed the observations in Fig 8. of the main paper.

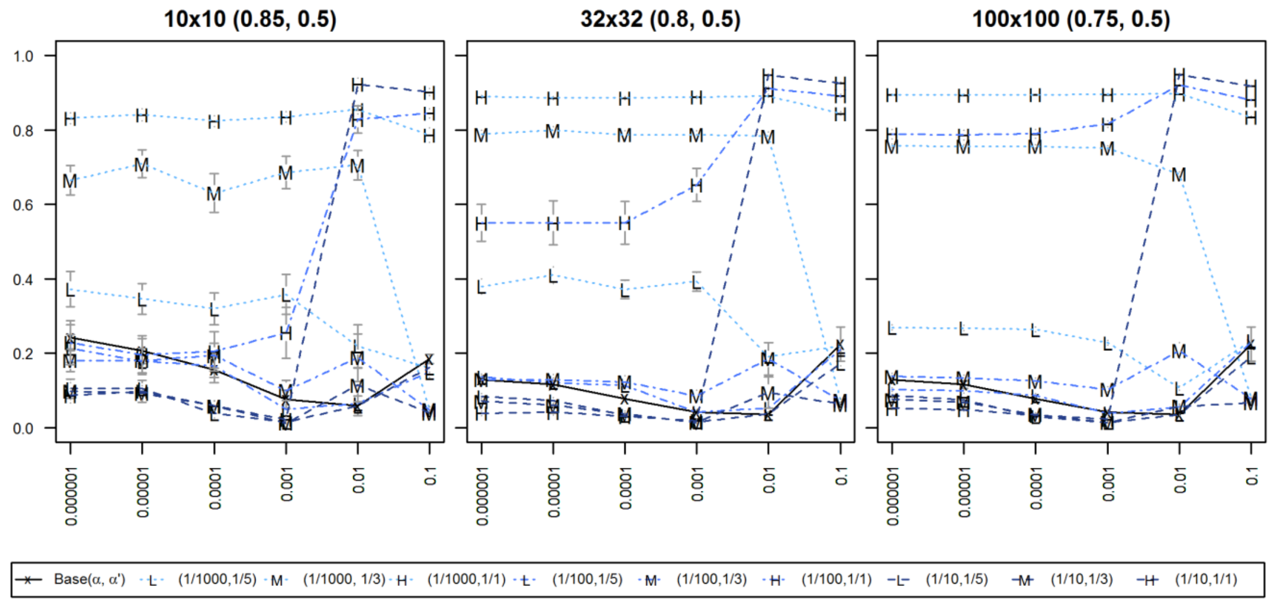

Fig. A. Cultural diversity for combinations of democracy and propaganda frequencies. X-axis displays levels of noise; Y axis displays normalized cultural diversity. Each line symbol denotes one combination of democracy and propaganda. 95% confidence intervals are displayed only when exceeding the size of the line symbol. Data points are averages of 50 replications per territory with 100,000 iterations per agent.
